# Trojan horse-inspired vaccine-like nanoparticle with enhanced lubrication and active-passive synergistic anti-inflammation for post-traumatic osteoarthritis treatment

**DOI:** 10.1016/j.bioactmat.2026.08.005

**Published:** 2026-08-12

**Authors:** Yinuo Yang, Jinna Ren, Gengtian Sun, Keyi Huang, Xin Zhao, Aleksandr Poliakov, Yuguang Wang, Hongyu Zhang

**Affiliations:** a State Key Laboratory of Tribology in Advanced Equipment, Department of Mechanical Engineering, Tsinghua University, Beijing, 100084, China; b Department of General Dentistry II & National Center for Stomatology, Peking University School and Hospital of Stomatology, Beijing, 100081, China; c Department of Applied Biology and Chemical Technology, The Hong Kong Polytechnic University, Hung Hom, Kowloon, Hong Kong SAR, 999077, China; d Polytechnic Institute, Sevastopol State University, Sevastopol, Russia

**Keywords:** Post-traumatic osteoarthritis, Hydration lubrication, Immune evasion, Trojan horse

## Abstract

Post-traumatic osteoarthritis (PTOA) is initiated by joint injury and progresses through the interaction of abnormal mechanical loading, oxidative stress, inflammation, and extracellular matrix degradation. Early intra-articular intervention is greatly limited by rapid clearance, insufficient lubrication, and dynamically evolving inflammatory microenvironment. Here, we developed a Trojan horse-inspired vaccine-like nanoparticle, PDMI, integrating immune-evasive retention, hydration lubrication, intrinsic reactive oxygen species (ROS) consumption, and ROS-responsive icariin (ICA) release. The hydrated exterior reduced macrophage recognition and interfacial shear, whereas the catechol groups and phenylboronic ester linkages consumed ROS and enabled microenvironment-responsive ICA release, respectively. PDMI reduced the coefficient of friction in simulated synovial fluid by approximately 52.07% at 3 N and 3 Hz and achieved coefficients of friction on the order of 0.001 under the tested microscale conditions. Intra-articular fluorescence imaging showed an apparent retention half-life of 22.93 days for PDMI, approximately 8.9-fold longer than that of free Cy5. In chondrocytes, PDMI reduced mitochondrial ROS, preserved mitochondrial membrane potential, restored NRF2-mediated antioxidant defense, and suppressed NF-κB/IκBα pathway activation and downstream inflammatory cytokine expression. In a 4-week rat PTOA model, the single intra-articular administration attenuated osteophyte formation and abnormal subchondral bone remodeling, reduced the OARSI score by more than 80%, decreased MMP-13 expression, and preserved glycosaminoglycan and Col2α levels. These findings position PDMI as a promising multifunctional intra-articular platform for early PTOA intervention and provide a foundation for its further translational development.

## Highlights

• A Trojan horse-inspired nanoparticle was developed with immune-evasive retention, hydration lubrication, and drug release.
• The nanoparticle established a progressive treatment paradigm that transitioned from lubrication enhancement to pharmacological intervention.
• The multi-dimensional therapeutic efficacy and biosafety of the nanoparticle were validated from both *in vitro* and *in vivo* tests.

## Graphical abstract

**Figure:**
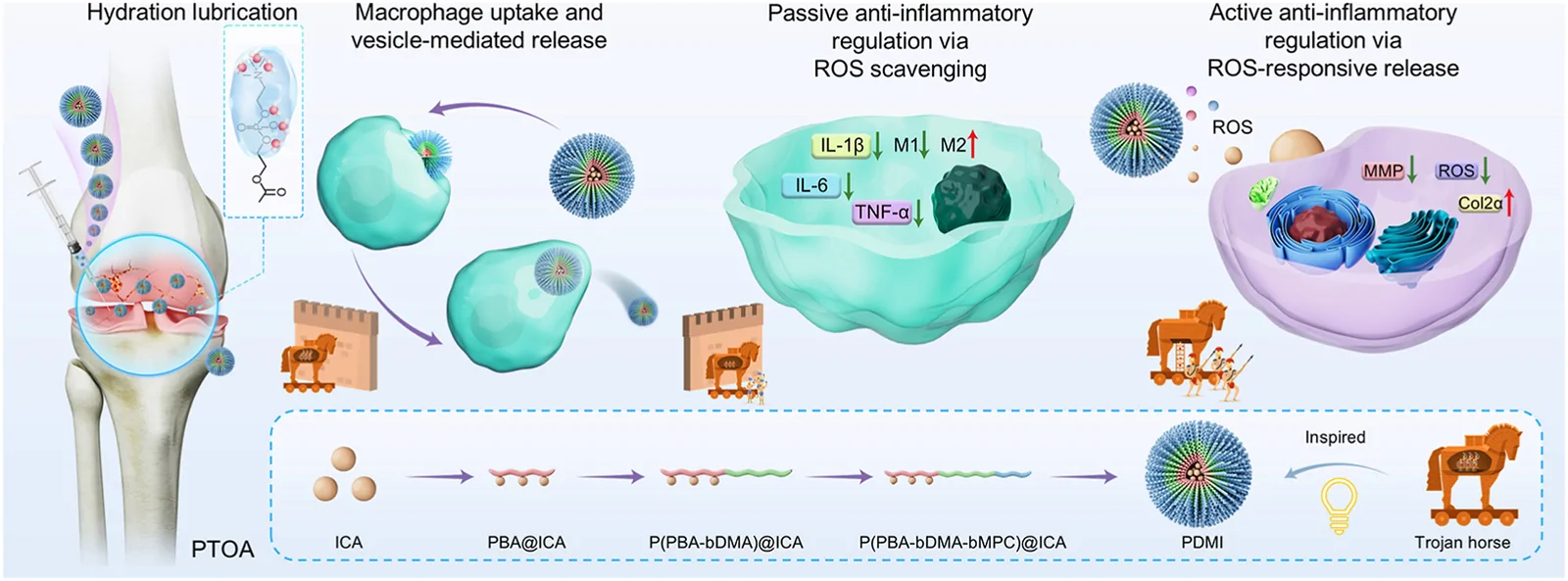

## 1 Introduction

Post-traumatic osteoarthritis (PTOA) develops after acute joint injuries and frequently affects young and physically active populations, including athletes and military personnel [[1], [2], [3]]. Although structural degeneration may not become clinically apparent immediately after trauma, mechanical instability, oxidative stress, synovial inflammation, and extracellular matrix disruption are initiated during the early post-injury period and progressively reinforce one another [[4], [5], [6]]. Once this self-amplifying pathological process is established, conventional treatments primarily relieve symptoms and rarely prevent cartilage degeneration [7,8]. The interval between joint injury and irreversible structural deterioration therefore represents an important therapeutic window in which early intervention may alter subsequent disease progression.

Effective intervention during this window requires simultaneous management of several interconnected barriers. Intra-articularly administered agents are rapidly diluted and cleared through synovial fluid turnover and macrophage-mediated recognition, resulting in insufficient local exposure [9,10]. At the same time, abnormal joint motion continuously increases interfacial friction and mechanical stimulation [[11], [12], [13]], while excessive ROS promotes mitochondrial dysfunction, inflammatory cytokine production, chondrocyte apoptosis, and matrix metalloproteinase expression [[14], [15], [16], [17]]. These biological and mechanical factors cannot be adequately addressed by a short-lived anti-inflammatory drug alone [[18], [19], [20]]. An effective early-intervention system should therefore remain within the joint, reduce friction at the cartilage interface, and adapt its therapeutic activity to the evolving oxidative and inflammatory microenvironment.

This requirement resembles the functional logic of the Trojan horse, in which a concealed functional component is protected by an external structure during entry and is subsequently deployed within the target environment. Translating this concept into a material system requires more than simple drug encapsulation. The external interface should reduce immune recognition and provide mechanical protection, whereas the internal chemistry should respond to pathological signals and expose the therapeutic component when needed. Zwitterionic materials such as phosphorylcholine, provide a suitable protective exterior because their strongly hydrated structure reduces nonspecific protein adsorption and macrophage recognition while also generating a low-shear lubrication layer [[21], [22], [23]]. For the responsive interior, the selected chemical units should both interact with excessive ROS and couple this reaction to therapeutic activation [24]. Dopamine-derived catechol groups are suitable ROS-reactive units because they can directly consume oxidants, whereas phenylboronic ester linkages undergo oxidative cleavage and simultaneously release conjugated therapeutic molecules [[25], [26], [27]]. The ROS-consuming reactions of these material components constitute the passive contribution to anti-inflammatory regulation, while the pharmacological activity of the released drug provides the active contribution. These effects may overlap temporally and collectively form an active-passive synergistic anti-inflammatory mechanism.

**Fig. 1:**
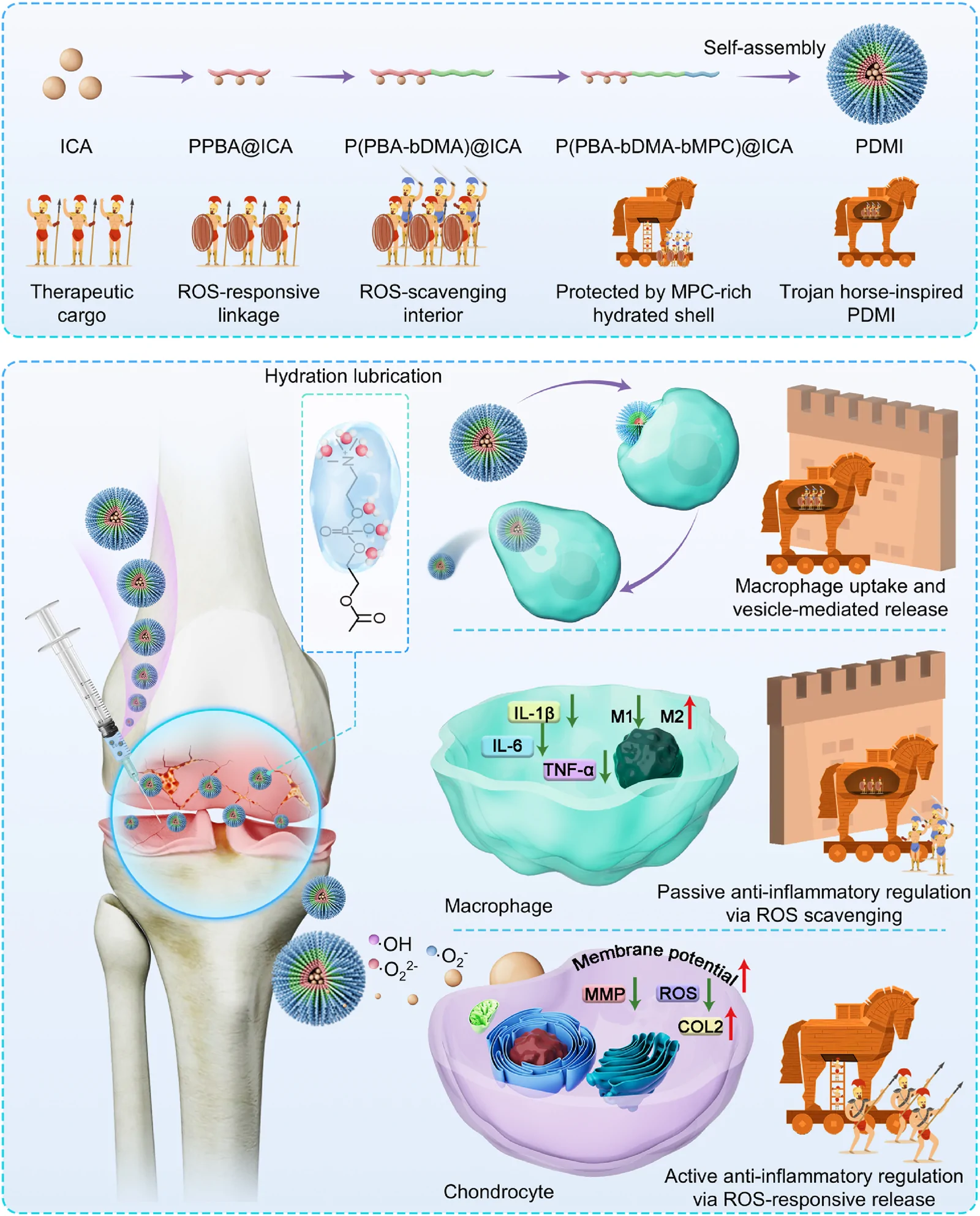
Schematic illustration of the construction and therapeutic functions of the Trojan horse-inspired PDMI nanoparticle for PTOA treatment. In the Trojan horse-inspired design, ICA served as the therapeutic cargo and was conjugated through ROS-responsive PBA@ICA linkages, while the DMA-containing region provided intrinsic ROS-scavenging activity and the MPC-rich hydrated exterior protected the system during intra-articular residence. The three functional domains of PDMI corresponded to three coordinated functions: the MPC-rich exterior reduced macrophage recognition and enhanced hydration lubrication, the DMA-containing interior consumed excessive ROS, and the ROS-responsive PBA@ICA unit released ICA for pharmacological anti-inflammatory regulation.

Based on this framework, we developed PDMI as a Trojan horse-inspired vaccine-like nanoparticle for early PTOA treatment. A ternary block copolymer composed of 3-(methacrylamido) phenylboronic acid, dopamine methacrylamide, and 2-methacryloyloxyethyl phosphorylcholine was synthesized to form PDM, followed by conjugation of icariin through ROS-responsive phenylboronic ester bonds. The MPC-rich hydrated exterior reduced macrophage recognition and enhanced interfacial lubrication, thereby protecting the system during intra-articular residence. Within the particle, DMA and phenylboronic ester chemistry provided intrinsic ROS-consuming activity, while ROS-triggered bond cleavage released ICA to further regulate inflammatory responses, mitochondrial function, and cartilage metabolism (Fig. 1). Thus, the Trojan horse inspiration was embodied by the coordinated sequence of external protection, concealed therapeutic loading, pathological signal recognition, and local drug deployment. The intra-articular retention, lubrication performance, ROS-responsive release, active-passive synergistic anti-inflammatory activity, and cartilage-protective efficacy of PDMI were systematically investigated.

## 2 Materials and methods

### 2.1 Materials and reagents

Dopamine hydrochloride, acrylyl chloride, and *N*,*N*-dimethylformamide (DMF) were purchased from Macklin Biochemical Technology Co., Ltd., Shanghai, China. Methacrylic anhydride, 4,4′-azobis(4-cyanovaleric acid) (ACVA), MPC, and FITC were purchased from J&K Scientific Ltd., Beijing, China. Sodium tetraborate decahydrate (Na_2_B_4_O_7_·10H_2_O) was purchased from Bide Pharmaceutical Technology Co., Ltd., Shanghai, China. Sodium bicarbonate (NaHCO_3_), magnesium sulfate (MgSO_4_), tetrahydrofuran, ethyl acetate, petroleum ether, and methanol were purchased from Titan Technology Co., Ltd., Shanghai, China. Sodium hydroxide (NaOH) was purchased from Boer Chemical Reagent Co., Ltd., Shanghai, China. Hydrochloric acid (HCl) and acetone were purchased from Tongguang Fine Chemical Company, Beijing, China. PBA and ICA were purchased from Konoscience Technology Co., Ltd., Beijing, China. Bis(carboxymethyl) trithiocarbonate, riboflavin, methionine, NBT, DPPH, and ABTS were purchased from Aladdin Bio-Chem Technology Co., Ltd., Shanghai, China. The water used in all of the experiments was deionized water with a resistivity of 18.2 MΩ cm. Paraformaldehyde, LPS, DAPI, DiO, anti-Col2α antibody, fluorescence-conjugated secondary antibody, and TRIzol reagent were purchased from Sigma-Aldrich Co., Ltd., Shanghai, China. ELISA kit was purchased from 4 A Biotech Co., Ltd., Beijing, China. CCK-8 kit was purchased from New Cell Molecular Biotech Co., Ltd., Suzhou, China. The live/dead viability/cytotoxicity assay kit was purchased from Keygen Biotech Co., Ltd., Nanjing, China. Penicillin-Streptomycin solution and fetal bovine serum were purchased from Aoqing Biotech Co., Ltd., Beijing, China.

### 2.2 Synthesis of PDM@ICA

First, DMA was synthesized as reported in the literature [28]. Afterward, the copolymer PDM used in the present work was synthesized via RAFT polymerization. The molar ratio of PBA, DMA, and MPC was maintained at 1:1:1, with ACVA as the reaction initiator and bis(carboxymethyl) trithiocarbonate as RAFT reagent. Specifically, PBA (205 mg, 1 mM), ACVA (2.8 mg, 0.01 mM), and RAFT reagent (11.2 mg, 0.05 mM) were dissolved in 50 mL of DMF under the protection of nitrogen, and the reaction was heated to 65°C for 24 h. Subsequently, DMA (221 mg, 1 mM) and ACVA (2.8 mg, 0.01 mM) were added into the mixed solution under nitrogen atmosphere and reacted at 65°C for 24 h. Next, MPC (295 mg, 1.00 mM) and ACVA (2.8 mg, 0.01 mM) in 50 mL of deionized water were added into the mixed solution and reacted at 65°C for 24 h under the protection of nitrogen. Finally, the solution was dialyzed against deionized water for 3 days using a dialysis bag (M_w_ = 1000 Da) and freeze-dried to give the copolymer product PDM.

The PDMI polymer grafting with drug was obtained through esterification reaction of phenylboronic acid and flavone. Generally, PDM (200 mg) and ICA (93.85 mg, 0.14 mM) were dissolved in a mixed solvent of deionized water and DMF at a volume ratio of 1:1 (25 mL each), followed by stirring at room temperature for 12 h. The resulting product was dialyzed in deionized water using a dialysis bag (M_w_ = 1000 Da) for 48 h, followed by freeze-drying.

### 2.3 Characterization of PDMI

The structural characterization of the polymer was performed using various analytical techniques. The ^1^H NMR spectroscopy was obtained by a 400 MHz NMR spectrometer (Avance III, Bruker, Germany). The infrared spectrum was measured employing a FTIR spectrometer (Horiba, Bruker, Germany). The particle size distribution of aggregates was analyzed using a size analyzer (Zetasizer Nano ZS, Malvern Instruments, UK), while the morphological characteristics were examined using a transmission electron microscope (HT7800, Hitachi, Japan). Additionally, XPS was performed using an X-ray photoelectron spectrometer (250XI, Thermo Fisher, UK). The elemental analysis was determined by a field emission transmission electron microscopy (TEM) combined with energy dispersive spectroscopy (JEM-2100F, JEOL, Japan). The molecular weight and distribution of the polymer were obtained by gel permeation chromatography (Vicotek TDA305, Malvern Instruments, UK).

### 2.4 *In vitro* drug grafting and release

The sustained release curve of PDMI was explored via *in vitro* drug grafting and release experiment, using traditional Chinese medicine ICA as the cargo. The calibration curve was initially obtained by recording the absorbance value of ICA in PBS across various concentrations, employing a spectrometer (UV-8000, Metash Instruments, China) at the wavelength of 269 nm. The calibration equation, Y = 7.37X-0.0057, where Y and X denoted the absorbance value and concentration of the solution, respectively, was used to calculate the grafting ratio, as shown below.

$$\text{Grafting}\;\text{ratio}=\frac{\text{weight}\;\text{of}\;\text{grafting}\;\text{ICA}}{\text{total}\;\text{weight}\;\text{of}\;\text{PDMI}}\times 100\%$$

The *in vitro* drug release kinetics of PDMI was determined using a dialysis method, which was performed for a duration of 48 h. Initially, PDMI (10 mg) was sonicated and uniformly dispersed in PBS (2 mL). The aqueous suspension was encapsulated into the dialysis bag with a molecular weight cutoff range of 8000-10000 Da. Subsequently, the dialysis bag was immersed in PBS (200 mL) with different concentrations of H_2_O_2_ (0, 1, 5, and 10 mM), simulating the inflammatory release medium, which was dialyzed at 37°C. At predetermined time intervals, the medium (2 mL) was removed, and then fresh solution (2 mL) was supplemented. The spectrophotometer was used to measure the absorbance value of the removed release medium at a wavelength of 269 nm, and the profile of cumulative drug release versus time was finally established according to the measurements.

### 2.5 ROS scavenging test

The scavenging efficiency of PDM and PDMI on the free radical was evaluated through colorimetric assay as described in previous studies [29,30]. For the NBT method, PDM and PDMI solutions at various concentrations were mixed with riboflavin (20 μM), methionine (12.5 mM), and NBT (75 μM), and dissolved in 2 mL of deionized water. The mixture was subsequently irradiated with ultraviolet light (365 nm) for 15 min. The absorption spectrum of the solution was measured using a UV spectrometer (UV-8000, Metash Instruments, China). Regarding the DPPH method, different concentrations of PDMI solution (2 mL) was evenly mixed with the ethanol solution of DPPH (0.1 mmol/L, 2 mL). Subsequently, the mixed solution was incubated in the dark at room temperature for 30 min, and then the absorbance of solution at 517 nm was measured using a multimode microplate reader (VICTOR Nivo 3S, PerkinElmer, USA). For the ABTS method, different concentrations of PDMI solution (2 mL) was mixed with the ethanol solution (2 mL) of ABTS (0.1 mmol/L) and potassium persulfate (0.0245 mmol/L) evenly. Subsequently, the mixed solution was incubated in the dark at room temperature for 5 min, and the absorbance of solution at 734 nm was measured using a multimode microplate reader (VICTOR Nivo 3S, PerkinElmer, USA). The scavenging efficiency was calculated based on the following formula, where A_i_ was the absorbance of solution with various concentrations of PDMI, and A_0_ was the absorbance of solution without PDMI.

$$\text{Scavenging}\;\text{efficiency}=\frac{A_{0}-A_{i}}{A_{0}}\times 100\%$$

### 2.6 Tribological test

To evaluate the lubrication performance of PDMI in an aqueous environment, a series of tribological tests were conducted employing AFM (Asylum Research, Oxford, USA). To minimize the influence of local surface chemistry at the microscale and reproduce a cartilage-like hydrated interface, both silica sheet and polystyrene microsphere were grafted with PMPC based on the method reported in a previous study [31]. During the experiment, the contact pressure was maintained within the range of 35-55 MPa, as calculated using the Hertz contact model. The scanning frequency was set between 1.0 and 3.0 Hz. For each experimental condition, three distinct regions with an area of 20 μm × 20 μm were randomly selected on the sample surface for testing. The average value was calculated to determine the COF under the corresponding loading conditions.

Additionally, the macroscopic lubrication performance was further evaluated using a multifunctional tribometer (UMT-5, Bruker, Germany) in reciprocating mode with a stroke length of 4 mm, sliding frequency of 1-3 Hz, and a test duration of 5 min under applied contact load of 1-3 N. Polytetrafluoroethylene ball (diameter = 8 mm) and silicon wafer were used as the upper and lower friction pairs, respectively. PDMI solutions (5 mg/mL) in simulated synovial fluid were used as the lubricant. The simulated synovial fluid consisted of 3 g/L sodium hyaluronate, 19 g/L bovine serum albumin, 11 g/L immunoglobulin G, and 0.1 g/L phospholipids dissolved in Ringer's solution. The COF was continuously recorded during the experiment, and the test was conducted in triplicate for each condition.

### 2.7 Cell culture

Primary chondrocytes were isolated from 3 to 5 days old male Sprague-Dawley rats. After euthanasia, the rat's body surface was disinfected with 75% ethanol. The knee joint capsule was opened, and the articular cartilage was harvested and digested overnight in 1 mg/mL of collagenase II (Invitrogen, Cat. 17101015). The following day, the residual tissue was removed, and the cells were cultured in Dulbecco's modified Eagle medium (Gibco, A011995) containing 10% fetal bovine serum (Gibco, 10270106). RAW264.7 cells were cultured in Dulbecco's modified Eagle medium supplemented with 10% heat-inactivated fetal bovine serum and 1% penicillin/streptomycin at 37°C in a 5% CO_2_ atmosphere.

### 2.8 CCK-8 assay

Chondrocytes and macrophages were seeded in 96-well plates at a density of 7 × 10^3^ cells/well and allowed to adhere and grow for 24 h. Afterward, different concentrations of PDMI were added to the wells, and the cells were incubated for an additional 24 h. Cell viability was assessed using the CCK-8 assay. Briefly, 10 μL of CCK-8 solution was added to each well, and the cells were incubated for 2 h. Absorbance was measured at the wavelength of 450 nm using a microplate reader (CMax Plus, Leica, Germany) to determine cell viability.

### 2.9 Hemolytic assay

The hemolytic assay of PMI, PDM, and PDMI was conducted utilizing fresh rat blood. Fresh blood samples underwent centrifugation at 3000 rpm for 15 min to isolate the red blood cells, which were subsequently washed three times with 0.9% saline solution. The diluted RBCs were then co-cultured with PMI, PDM, and PDMI at 37°C for 4 h. A positive control, representing 100% hemolytic efficiency, was established using 0.1% Triton X-100. The negative control was PBS. Following centrifugation at 3000 rpm for 15 min, the absorbance at the wavelength of 570 nm was quantified using the microplate reader (CMax Plus, Leica, Germany). The hemolysis rate was calculated by comparing the absorbance values of the experimental samples with those of the positive control.

### 2.10 Live/dead staining assay

Cell viability was assessed using a live/dead staining kit (Beyotime, China). RAW264.7 macrophages and chondrocytes were seeded in 6-well plates at a density of 1 × 10^4^ cells/well and treated with PMI, PDM, or PDMI for 24 h at 37°C in a 5% CO_2_ incubator. Subsequently, the culture medium was removed, and the cells were thoroughly washed with PBS. Calcein-AM was used to stain live cells, and propidium iodide (PI) was used to stain dead cells. The staining solution was added to each well and incubated at 25°C for 30 min, followed by observation using confocal laser scanning microscope CLSM (FV3000, Olympus, Japan).

### 2.11 Chondrocyte uptake experiment

Rat primary chondrocytes were seeded in 6-well plates at a density of 1 × 10^4^ cells/well and incubated for 24 h. Afterward, the cells were then co-cultured with PMI, PDM, or PDMI for 4 h, after which they were fixed with 4% paraformaldehyde. The cytoskeleton of chondrocytes was labeled with phalloidin fluorescence, and the cell nucleus was stained with Hochest. Subsequently, CLSM (FV3000, Olympus, Japan) was used to examine the acquired cell images, and FCM (Accuri C6, BD Biosciences, USA) was used to evaluate the uptake efficiency of chondrocytes.

### 2.12 Immune escape experiment

RAW264.7 macrophages were seeded in 6-well plates at a density of 1 × 10^4^ cells/well and incubated for 24 h. The cells were co-cultured with PMI, PDM, or PDMI for 48 h and fixed with 4% paraformaldehyde. Subsequently, the cytoskeleton of macrophages was labeled with phalloidin fluorescence, and the cell nucleus was stained with Hochest. The acquired cell images were examined using CLSM (FV3000, Olympus, Japan), and the immune escape efficiency was evaluated using FCM (Accuri C6, BD Biosciences, USA). Furthermore, after co-culturing PDMI with RAW264.7 macrophages for 48 h, the culture supernatant was collected and the vesicles derived from the macrophages were enriched through centrifugation. The vesicles were observed using a transmission electron microscope (HT7800, Hitachi, Japan).

### 2.13 RNA sequencing and DEGs analysis

Total RNA was extracted using TRIzol reagent (Invitrogen). RNA purity was determined and quantified using a NanoDrop 2000 spectrophotometer. Transcriptome sequencing and analysis were performed with the assistance of Baipu Biotechnology, Shanghai, China. RNA sequencing was performed using three independent biological replicates for each experimental group. DEGs were evaluated employing KEGG enrichment analysis, with p-value < 0.05, q-value < 0.05, and fold change > 1.2. Consequently, the biological functions and pathways significantly influenced by these differential transcripts were determined. The unsupervised hierarchical clustering of the differential transcripts was performed, and the heat map was generated to visualize the expression patterns across different samples. Additionally, the volcano plot and MA plot were established to visualize the distribution of DEGs, highlighting gene significance, fold changes, and adjusted p-values. Finally, the principal component analysis was conducted to reduce dimensionality and describe the differences between various groups.

### 2.14 Western blot

Western blot was performed to evaluate the regulatory effects of PDMI on antioxidant and anti-inflammatory signaling pathways in chondrocytes. Briefly, primary rat articular chondrocytes subjected to different treatments were lysed using RIPA lysis buffer supplemented with 1% protease inhibitor cocktail (Solarbio, China), and total proteins were extracted. The protein samples were separated by 10% sodium dodecyl sulfate-polyacrylamide gel electrophoresis and subsequently transferred onto polyvinylidene fluoride membranes. The membranes were blocked with 5% non-fat milk at room temperature for 2 h and then incubated overnight at 4°C with primary antibodies against NRF2 (1:1000, Cell Signaling Technology, USA), P65 (1:4000, Abiowell Technology, USA), p-P65 (1:1000, Cell Signaling Technology, USA), p-IκBα (1:1000, Cell Signaling Technology, USA), IκBα (1:1000, Cell Signaling Technology, USA), and GAPDH (1:8000, Proteintech, USA), followed by incubation with the corresponding secondary antibodies. Finally, the protein bands were visualized using an enhanced chemiluminescence detection kit.

### 2.15 Reverse transcription quantitative polymerase chain reaction (RT-qPCR) analysis

RT-qPCR was performed to determine the mRNA expression levels of the inflammation-related genes TNF-α, IL-6, and IL-1β. Total RNA was extracted from cells subjected to different treatments using a commercial RNA isolation kit (Beyotime, China), and the RNA concentration and quality were assessed using a NanoDrop One spectrophotometer (Thermo Scientific, USA). The extracted RNA was subsequently reverse-transcribed into cDNA using cDNA Synthesis SuperMix (YEASEN, China). The qPCR reaction mixture contained the synthesized cDNA, qPCR Master Mix (YEASEN, China), gene-specific primers, and nuclease-free water, and amplification was performed using an Archimed real-time quantitative PCR system (ROCGENE, China). Relative gene expression was calculated using the 2^−ΔΔCt^ method and normalized to GAPDH as the internal reference gene.

### 2.16 *In vitro* mitochondrial ROS detection

Mitochondrial ROS in chondrocytes was assessed by MitoSOX assay. Chondrocytes were seeded in 6-well plates at a density of 1 × 10^4^ cells/well and incubated for 24 h. To simulate the PTOA microenvironment, 100 μM H_2_O_2_ was added to each well, and the cells were incubated for 8 h. After PBS washing, PMI, PDM, or PDMI was added, and the cells were incubated for an additional 24 h. Subsequently, 10 μL MitoSOX solution (Sigma-Aldrich Co., Ltd., Shanghai, China) was added after PBS washing, and the cells were incubated for 30 min. The *in vitro* mitochondrial ROS was evaluated using CLSM (FV3000, Olympus, Japan).

### 2.17 *In vitro* mitochondrial membrane potential

Mitochondrial membrane potential in chondrocytes was characterized employing JC-1 assay. Chondrocytes were seeded in 6-well plates at a density of 1 × 10^4^ cells/well and incubated for 24 h. To simulate the PTOA microenvironment, 100 μM H_2_O_2_ was added to each well, and the cells were incubated for 8 h. After PBS washing, PMI, PDM, or PDMI was added, and the cells were incubated for an additional 24 h. 10 μL JC-1 solution (Sigma-Aldrich Co., Ltd., Shanghai, China) was added after PBS washing, and the cells were incubated for 30 min. CLSM (FV3000, Olympus, Japan) was used to assess *in vitro* mitochondrial membrane potential in chondrocytes.

### 2.18 Col2α expression of chondrocytes

Chondrocytes were seeded in 6-well plates at a density of 1 × 10^4^ cells/well and then incubated for 24 h. To simulate the PTOA microenvironment, 100 μM H_2_O_2_ was added to each well, and the cells were incubated for 8 h. After PBS washing, PMI, PDM, or PDMI was added, and the cells were incubated for an additional 24 h. The chondrocytes were then fixed with 4% paraformaldehyde for 20 min and washed with PBS. To allow antibody penetration, the cells were permeabilized with 0.1% Triton X-100 solution for 30 min. Non-specific binding was blocked with 10% normal goat serum at 25°C for 1 h. Primary antibody against Col2α (1:2000) was applied, and the cells were incubated overnight at 4°C. The following day, the cells were incubated with fluorescently labeled secondary antibodies (1:200) in the dark for 1 h, followed by staining with Hochest and phalloidin for 15 min. The immunofluorescence images were captured using CLSM (FV3000, Olympus, Japan), and the expression of Col2α in chondrocytes was evaluated using FCM (Accuri C6, BD Biosciences, USA).

### 2.19 *In vitro* chondrocyte apoptosis analysis

Chondrocytes were seeded in 6-well plates at a density of 1 × 10^4^ cells/well and then incubated for 24 h. Afterward, 100 μM H_2_O_2_ was added to each well, and the cells were incubated for 8 h. After PBS washing, PMI, PDM, or PDMI was added, and the cells were incubated for an additional 24 h. CCK-8 assay was used to evaluate chondrocyte viability, and FCM (Accuri C6, BD Biosciences, USA) was used to assess the apoptosis rate of chondrocytes.

### 2.20 *In vitro* macrophage apoptosis and polarization

Initially, RAW264.7 macrophages were stimulated with LPS for 24 h to induce the M1 phenotype. The macrophages were then treated with PDMI for 24 h. FCM (Accuri C6, BD Biosciences, USA) was utilized to assess the apoptosis rate of macrophages. After treatment, the cells were washed three times with PBS and blocked with 1% bovine serum albumin. Subsequently, the cells were incubated at room temperature with anti-CD86-FITC (M1 marker, 1:100) and anti-CD86-Alexa Fluor 647 (M2 marker, 1:100) for 1 h. Macrophage polarization was analyzed by FCM (Accuri C6, BD Biosciences, USA).

### 2.21 Dual-fluorescence colocalization analysis of PDMI structural integrity

To determine whether the detected fluorescence signal predominantly originated from intact PDMI nanoparticles, PDMI was dual-labeled with FITC and maleimide-Cy5. The spatial distributions of the FITC and Cy5 fluorescence signals were then examined by CLSM, and their colocalization was evaluated using merged images. The degree of overlap between the FITC and Cy5 signals was used to assess whether the detected fluorescence was primarily associated with intact PDMI nanoparticles rather than free fluorescent dyes or individual degradation fragments.

### 2.22 Macrophage exocytosis assay

FITC-labeled PDMI nanoparticles were incubated with RAW264.7 macrophages for 6 h to allow sufficient cellular internalization. After incubation, the nanoparticle-containing medium was removed, and the cells were washed three times with PBS to eliminate noninternalized nanoparticles and particles adsorbed onto the cell surface. The cells were then randomly divided into two groups. One group was cultured in fresh complete medium without nanoparticles, whereas the other group was cultured in fresh complete medium containing GW4869 (10 μM) but no nanoparticles. After an additional 6, 12, or 24 h of incubation, the cells and corresponding culture supernatants were collected. The cells were washed with PBS, detached by enzymatic digestion or gentle pipetting, and analyzed by flow cytometry to determine intracellular FITC fluorescence. The culture supernatants were centrifuged to remove cellular debris, and the extracellular FITC fluorescence was subsequently measured by flow cytometry to evaluate the exocytosis of PDMI from RAW264.7 macrophages and the effect of GW4869 on this process.

### 2.23 Cytocompatibility evaluation of GW4869

To exclude the possibility that passive fluorescence release resulted from GW4869-induced cellular injury or death, the effect of GW4869 (10 μM) on RAW264.7 macrophage viability was evaluated using a cell viability assay. After GW4869 treatment, cell viability was measured according to the manufacturer's instructions and compared with that of the control group.

### 2.24 Anti-inflammatory assessment

RAW264.7 macrophages were cultured in 6-well plates at a density of 1 × 10^4^ cells/well and incubated at 37°C in 5% CO_2_ for 24 h. The culture medium containing LPS (5 μg/mL) was added to each well, except for the control group, to activate macrophages to produce inflammatory cytokines. After 8 h of activation, the cells were washed with PBS. Subsequently, the culture medium containing PMI, PDM, or PDMI was added to the wells. After 24 h of incubation, the inflammatory cytokines TNF-α, IL-6, and IL-1β were quantified by ELISA according to the manufacturer's instructions.

Besides, chondrocytes were cultured in 6-well plates at a density of 1 × 10^4^ cells/well and incubated at 37°C in 5% CO_2_ for 24 h. Afterward, 100 μM H_2_O_2_ was added to each well except for the control group. After 8 h of activation, the cells were washed with PBS. Subsequently, the culture medium containing PMI, PDM, or PDMI was added to the wells. After 24 h of incubation, MMP-13 expression was quantified by ELISA according to the manufacturer's instructions.

### 2.25 *In vivo* fluorescence intensity detection

To evaluate the retention time of PDMI *in vivo*, rats were anesthetized, and hair around the knee joint was carefully removed. Afterward, 30 μL of Cy5 or Cy5-labeled PDMI formulation containing the same amount of fluorescent dye was injected into the joint cavity of the knee. The fluorescence imaging was immediately performed, and further imaging was conducted on day 0, 3, 6, 9, 12, 15, 18 and 21 using a small animal imaging system (FluorVivo, Toronto, Canada).

### 2.26 Surgical PTOA model in rats

All experimental procedures involving animals were performed in accordance with the guidelines approved by the Animal Care and Welfare Committee of Peking University School of Stomatology (BDKQ-202510160923). The PTOA model in rats was established using ACLT and pMMx. The rats were randomly divided into five groups (each group n = 4) including (i) sham surgery, (ii) ACLT + pMMx and PBS treatment, (iii) ACLT + pMMx and PMI treatment, (iv) ACLT + pMMx and PDM treatment, and (v) ACLT + pMMx and PDMI treatment. A running training was initiated post-surgery to accelerate the progression of PTOA. For treatment groups, the intra-articular injection was administered on day 2 post-surgery. On day 30, all the rats were euthanized for the following characterizations.

### 2.27 Micro-CT analysis

After the rats were euthanized, the knee joints were excised. The samples were fixed in 4% paraformaldehyde for 48 h and then examined using Micro-CT (Inveon Scanners, Siemens, Germany). The subchondral bone was reconstructed and analyzed employing the corresponding software. The morphometric evaluation of tibial subchondral bone was performed based on three-dimensional data, including parameters such as BV/TV, BS/BV, Tb.N, Tb.Sp, and Tb.Th.

### 2.28 Histological evaluation

Knee joint tissues from the sham, PTOA, PMI, PDM, and PDMI groups were collected and fixed in 4% paraformaldehyde for 48 h. The samples were then decalcified in 10% ethylene diamine tetraacetic acid at 37°C using a rapid tissue processor (ASP300S, Leica, Germany), embedded in paraffin, and sectioned into 5 μm thick continuous slices using a rotary microtome (RM2235, Leica, Germany). Sections were stained with H&E, SO/FG, and TB staining. Histological analysis was performed using a digital pathology scanner (Aperio AT2, Leica, Germany) to assess joint morphology and inflammation scores.

### 2.29 Malondialdehyde (MDA) level

MDA levels in articular cartilage tissues were measured using an MDA assay kit (Beyotime, China) according to the manufacturer's instructions. Briefly, cartilage tissues were supplemented with nine volumes of physiological saline at a tissue weight to solution volume ratio of 1:9 g/mL and mechanically homogenized under low-temperature conditions. The homogenates were centrifuged at 12,000 rpm for 10 min, and the resulting supernatants were collected for analysis. The MDA working solution was prepared by thoroughly mixing the thiobarbituric acid dilution buffer, TBA stock solution, and antioxidant reagent. The working solution was then added to each sample, followed by incubation at ≥95°C for 15 min. After cooling to room temperature, the samples were centrifuged at 3000 rpm for 10 min, and the absorbance of the resulting supernatants was measured at 532 nm.

### 2.30 Immunohistochemical analysis

To further evaluate the therapeutic effect of PDMI on PTOA, immunohistochemistry was performed to assess the expression levels of Col2α and MMP-13 proteins in knee joint cartilage tissue. After dewaxing, tissue sections were incubated overnight at 4°C with primary antibodies against Col2α (1:100) and MMP-13 (1:100). Following PBS washing, the sections were incubated with secondary antibodies (1:100) at 25°C for 1 h. Following incubation, the sections were immersed in diaminobenzidine solution and counterstained with hematoxylin. The images were observed using a digital pathology scanner (NanoZoomer-SQ, Hamamatsu, Japan). Quantitative analysis was performed using ImageJ software.

### 2.31 Immunofluorescence analysis

Immunofluorescence staining was performed to evaluate macrophage infiltration and polarization in rat knee joint tissues. Paraffin-embedded joint sections were deparaffinized, rehydrated, and subjected to antigen retrieval, followed by permeabilization with 0.1% Triton X-100 and blocking with 5% normal goat serum for 1 h at room temperature. The sections were then incubated overnight at 4°C with primary antibodies against F4/80, iNOS, or Arg-1, followed by incubation with the corresponding fluorescence-conjugated secondary antibodies for 1 h at room temperature in the dark. Cell nuclei were counterstained with DAPI, and fluorescence images were acquired using CLSM. The relative fluorescence intensity or positive fluorescence area of each marker was quantified using ImageJ software.

### 2.32 Biocompatibility

To evaluate the *in vivo* biocompatibility of PDMI, H&E staining was performed on the major organs, including heart, liver, spleen, lung, and kidney, of rats at the experimental endpoint. Additionally, whole blood and serum samples were collected from each group of rats for quantitative analysis of complete blood count and blood biochemistry.

### 2.33 Statistical analysis

All the experiments were independently repeated at least three times, and representative data were shown. Data were presented as mean ± standard deviation (SD). For multiple-group comparisons, residual normality and homogeneity of variance were assessed using the Shapiro-Wilk and Brown-Forsythe tests, respectively. Data satisfying these assumptions were analyzed using one-way ANOVA followed by Tukey's multiple-comparison test, while appropriate alternative tests including Welch's ANOVA or nonparametric tests, were applied when the assumptions were not met. A value of p < 0.05 was considered statistically significant. The statistical significance was indicated as *p < 0.05, **p < 0.01, ***p < 0.001, and ****p < 0.0001.

## 3 Results

### 3.1 Synthesis and characterization of the PDMI

**Fig. 2:**
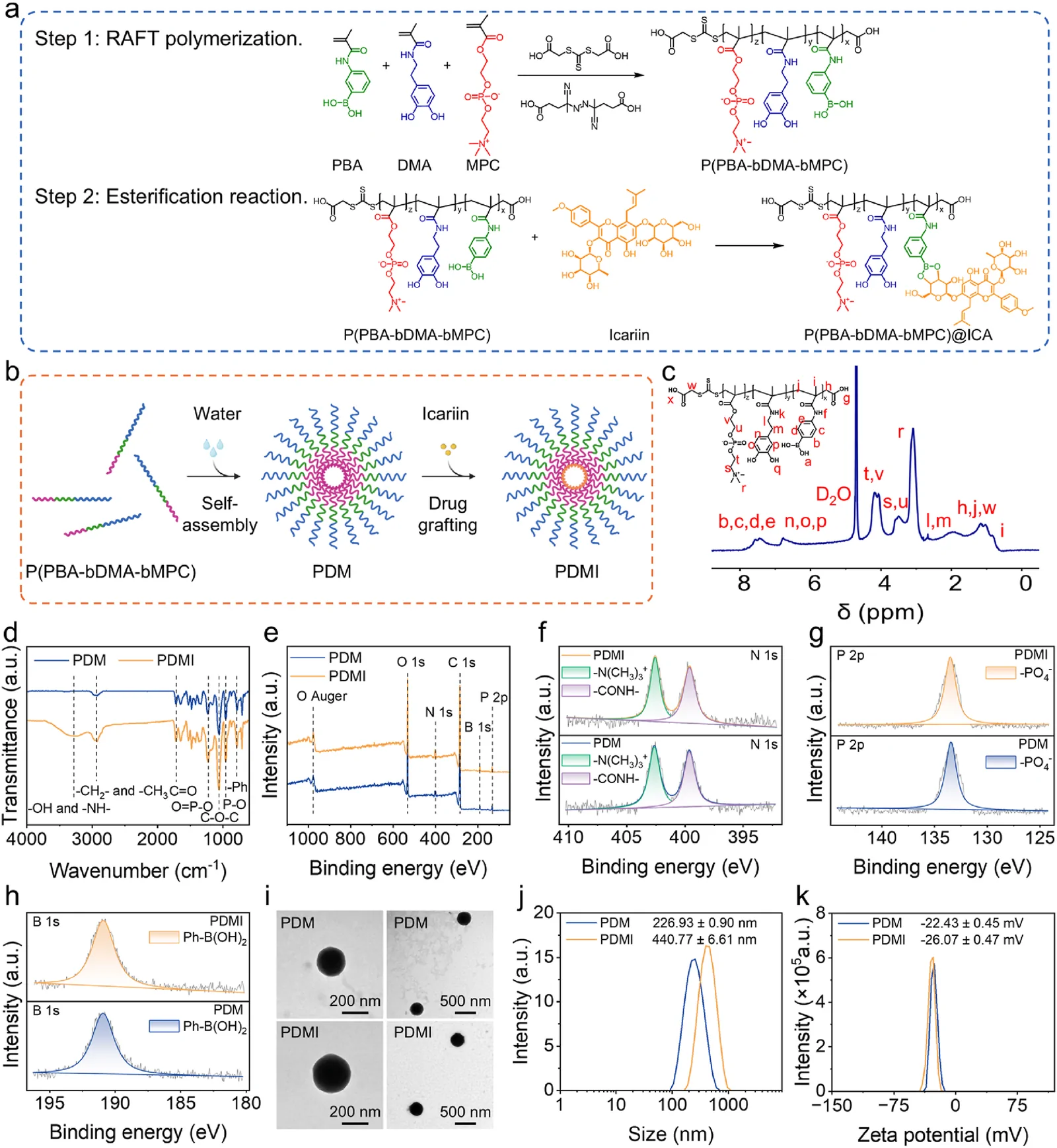
Synthesis and characterization of PDMI. (a) Synthesis route of PDMI. (b) Schematic diagram of self-assembly of PDMI. (c) ^1^H NMR spectrum of ternary polymer PDM. (c) FTIR spectrum. (d) XPS full spectrum of PDMI. (f-h) High-resolution narrow spectra of N 1s, P 2p and B 1s, respectively. (i) Representative TEM images of PDM and PDMI. (j) Size distribution of PDM and PDMI. (k) Zeta potential distribution of PDM and PDMI.

The ternary block copolymer P(PBA-bDMA-bMPC) (abbreviated as PDM) was successfully synthesized via reversible addition-fragmentation chain transfer (RAFT) polymerization, as depicted in Fig. 2a. Then, traditional Chinese medicine (ICA) was grafted into PDM via esterification reaction to obtain PDMI. The theoretical ICA feed amount was determined according to the 1:1 stoichiometric reaction between the phenylboronic acid groups in PDM and the available diol-containing hydroxyl groups of ICA, while the actual ICA content in PDMI was governed by the experimentally determined conjugation efficiency. Its molecular structure displayed clear amphiphilicity, with PBA and DMA constituting the hydrophobic segment and MPC acting as the hydrophilic segment, thus leading to the formation of nanoparticle through self-assembly, named as PDMI (Fig. 2b). Proton nuclear magnetic resonance (^1^H NMR) spectrum of PDM (Fig. 2c) showed typical proton signals from the benzene ring (PBA: *δ* = 7.56, 7.44 ppm; DMA: *δ* = 6.87, 6.72 ppm) and characteristic peaks of MPC (methylene: *δ* = 4.18-4.06 ppm, methyl: *δ* = 3.51, 3.08 ppm), confirming the integrity of the block structure. The ^1^H NMR spectrum of PDMI further exhibited characteristic proton signals attributable to ICA (Fig. S1), while the major signals of the PDM backbone remained well preserved, supporting the successful conjugation of ICA without appreciable disruption of the polymer structure. FTIR spectroscopy showed an enhanced broad absorption band at approximately 3400 cm^−1^ after ICA conjugation, which was attributed to changes in the hydroxyl chemical environment (Fig. 2d). X-ray photoelectron spectroscopy (XPS) further elucidated chemical mechanism underlying the grafting process (Fig. 2e). The N 1s spectrum revealed the presence of quaternary ammonium nitrogen (-N(CH_3_)_3_^+^, 402.5 eV) and amide nitrogen (-CONH-, 399.6 eV) in MPC, consistent with the designed molecular structure (Fig. 2f). The stable binding energy of the P 2p peak (133.5 eV) confirmed the core polymer structure remained intact (Fig. 2g). Additionally, the increase of B 1s binding energy (from 190.8 to 191.4 eV, Fig. 2h) was attributed to the formation of boronic ester bond between the boronic acid group and ICA.

Representative TEM images present in Fig. 2i showed that the PDM and PDMI were both spherical, uniformly shaped, and exhibited good monodispersity. Owing to the amphiphilic block architecture, the relatively hydrophobic PBA, DMA, and ICA-containing segments tended to associate in the aqueous environment, whereas the hydrophilic MPC segments extended toward the surrounding water phase. This molecular arrangement promoted the curvature and closure of the polymer chains into a vesicle-like nanostructure comprising a polymer-rich shell and an internal aqueous cavity. Consistent with this proposed hollow-like architecture, EDS elemental mapping showed approximately uniform distributions of C, B, N, and O, while the P signal associated with the MPC segments was preferentially distributed toward the particle periphery (Fig. S2). Dynamic light scattering measurements revealed an increase in the average hydrated particle size from 226.93 to 440.77 nm after ICA grafting, which was attributed to enhanced hydrophobic association (Fig. 2j–k). Furthermore, PDMI maintained narrow particle size distribution at pH 5.5, 6.5, and 7.4, which represented acidic inflammatory, mildly acidic pathological, and physiological synovial environments, respectively, without obvious particle size increase, peak broadening, or multimodal distribution (Fig. S3). This result indicated that PDMI preserved good colloidal stability across physiologically relevant pH conditions. However, the value of surface zeta potential did not demonstrate significant change, shifting slightly from −12.3 to −11.7 mV, which was conducive to evading recognition by macrophages through electrostatic interaction.

### 3.2 Stimuli-responsive drug release behavior

**Fig. 3:**
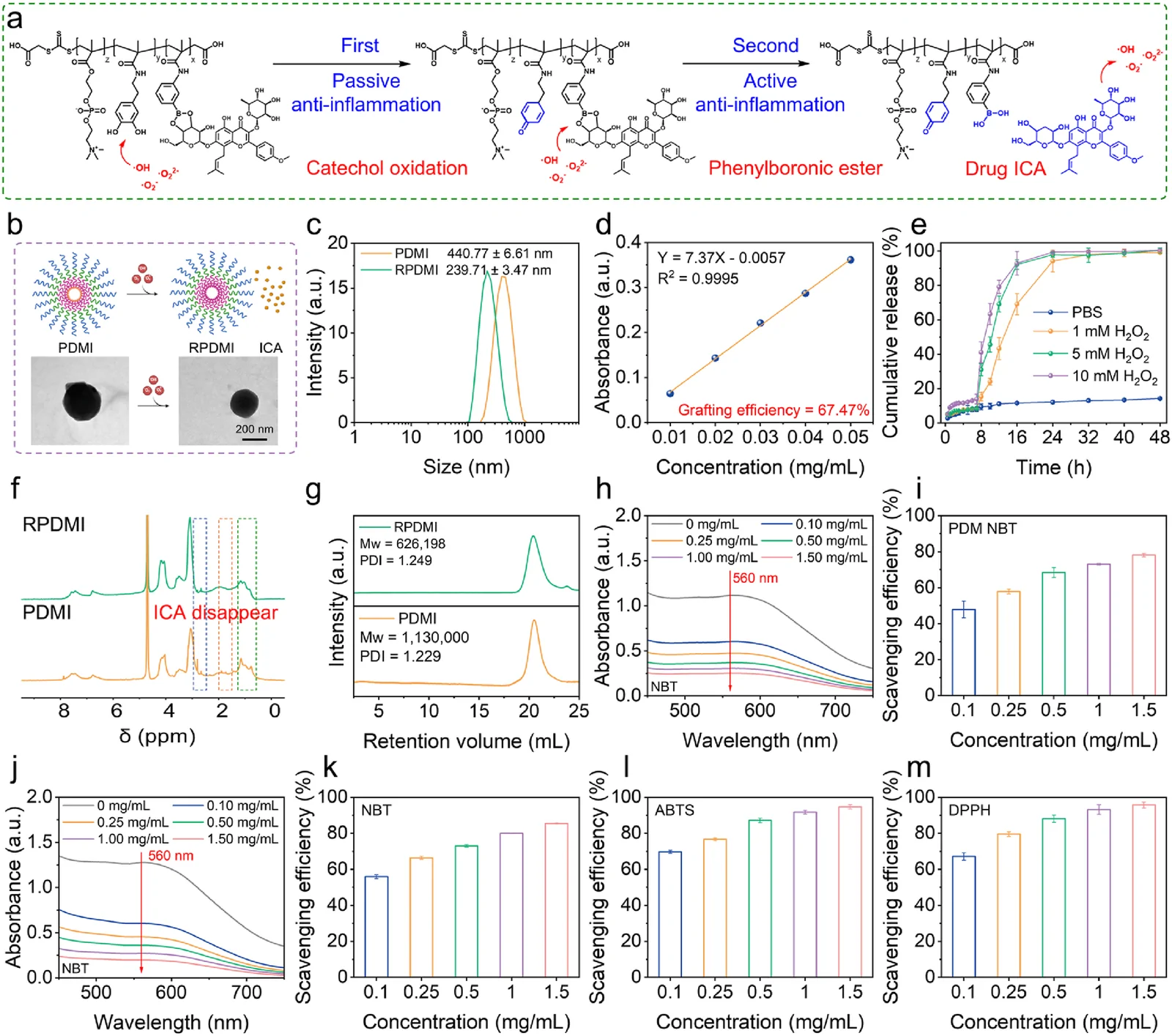
Characterization of ROS-responsive drug release profile and ROS scavenging efficiency of PDMI. (a) The chemical reaction equation between the PDMI and ROS, achieving passive anti-inflammation and active anti-inflammation. (b) Schematic diagram of the ROS-responsive drug release mechanism and representative TEM images of PDMI and RPDMI. (c) Size distribution of PDMI and RPDMI. (d) Standard curve of absorbance of ICA under different concentrations in phosphate buffered saline (PBS). (e) Cumulative drug release curves under different conditions. The data were presented as mean ± SD, n = 3. (f) ^1^H NMR spectra of PDMI before and after ROS response. (g) GPC spectra of PDMI before and after ROS response. (h-i) UV-vis absorbance spectra and scavenging efficiency of PDM determined by the NBT assay. The data were presented as mean ± SD, n = 3. (j-k) UV-vis absorbance spectra and scavenging efficiency of PDMI determined by the NBT assay. (l-m) Scavenging efficiency of PDMI determined by ABTS and DPPH assay, respectively. The data were presented as mean ± SD, n = 3.

The anti-inflammatory function of the PDMI arose from two aspects, as shown in Fig. 3a. Firstly, the catechol structure of DMA in the PDMI functioned as a gatekeeper, serving as the first barrier by reacting with ROS generated in the PTOA microenvironment and being oxidized into quinone, thereby exerting a passive anti-inflammatory effect. Secondly, the phenylboronic acid ester bond formed by PBA and ICA acted like a shield in a soldier's hand, reacting with excessive ROS to experience cleavage and eliminating excessive ROS. Simultaneously, the drug ICA serving as a soldier was released and subsequently invaded the inflamed cells, thereby achieving an active anti-inflammatory function. Since the drug release did not alter the hydrophilic-hydrophobic nature of the copolymer molecule, the nanoparticle maintained a uniform spherical morphology, abbreviated as RPDMI (Fig. 3b). After ROS response, the hydrated size of RPDMI decreased to 239.71 nm, due to the release of the hydrophobic drug ICA, which made the nanoparticle structure more compact (Fig. 3c). The drug grafting efficiency was quantitatively assessed according to an absorbance versus concentration standard curve (R^2^ = 0.9995, Fig. 3d) measured based on UV-visible spectrophotometry, revealing a grafting rate of 67.47%, which was higher than that of the non-covalent drug loading system (usually 20-30% [[32], [33], [34]]).

In the inflammatory pathological microenvironment, ROS level could be markedly elevated to the range of 1-100 mM. Accordingly, corresponding concentrations of H_2_O_2_ were used in this study to mimic such pathophysiological oxidative stress conditions. The phenylboronic ester bonds in the PDMI were specifically cleaved in response to these elevated ROS level, thereby enabling controlled drug release at the lesion site. As displayed in Fig. 3e, the release behavior of the PDMI under simulated ROS condition exhibited a notable feature. Specifically, in the absence of ROS, ICA was scarcely released (∼14% at 48 h). By contrast, in H_2_O_2_-containing media, ICA was released following a lag phase of approximately 8 h. This behavior was attributed to dopamine, which functioned against ROS permeation during the initial 8 h. Once dopamine was depleted, drug release was triggered accordingly. The breakdown of phenylboronic ester bond was like a protective shield to scavenge excessive ROS while releasing ICA as a soldier to enter the inflamed cells to alleviate disease symptoms. Consistently, after 8 h, the release medium exhibited the characteristic absorption peak of ICA at approximately 269 nm (Fig. S4), whose intensity gradually increased with release time. Meanwhile, the PDMI solution progressively became lighter without obvious precipitation or macroscopic aggregation (Fig. S5). Over 48 h, the cumulative release approached nearly 100%, and the release rate increased with rising H_2_O_2_ concentration. Besides, ^1^H NMR (Fig. 3f) and gel permeation chromatography (GPC) (Fig. 3g) further confirmed that the ICA characteristic peak disappeared and the molecular weight of polymer chain decreased slightly after drug release, indicating bond cleavage during the release process.

### 3.3 ROS scavenging efficiency

To evaluate the ROS-scavenging capability, we employed three methods to quantify the efficiency of free radical clearance. Initially, the nitroblue tetrazolium (NBT) method was utilized to quantify the scavenging efficiency of superoxide anion (O_2_·^-^). As shown in Fig. 3h–k, the PDMI exhibited a clearance efficiency of 86% at the concentration of 1.5 mg/mL, and this result outperformed the unloaded system PDM (only passive anti-inflammation), which showed 72% clearance efficiency. Additionally, both the 1,1-diphenyl-2-nitrobenzhydrazine (DPPH) and 2,2′-azinobis (3-ethylbenzothiazoline-6-sulfonic acid) (ABTS) methods were used to further assess the *in vitro* antioxidant properties of the PDMI. With the increase in the concentration of the PDMI, the clearance ability against DPPH·and ABTS·radicals enhanced obviously (Fig. 3l–m). When the concentration of the PDMI reached 1 mg/mL, the clearance efficiency against DPPH·radicals exceeded 90%, which indicated that the PDMI had a strong free radical clearance ability. Similar to the result of the DPPH radicals, at the concentration of 1.5 mg/mL, the clearance efficiency against ABTS^+^ reached approximately 95%. It was precisely because the PDMI had both active anti-inflammatory and passive anti-inflammatory dual effects and was endowed with outstanding micro-environmental regulation capability, especially in scavenging ROS. These results indicated that the PDMI had excellent antioxidant potential *in vitro*, which would be further confirmed through the cell and animal experiments that the PDMI could effectively eliminate the disease-related ROS in the pathological microenvironment of PTOA, thus more accurately reflecting its anti-inflammatory and therapeutic efficacy in complex biological systems.

### 3.4 Lubrication performance

**Fig. 4:**
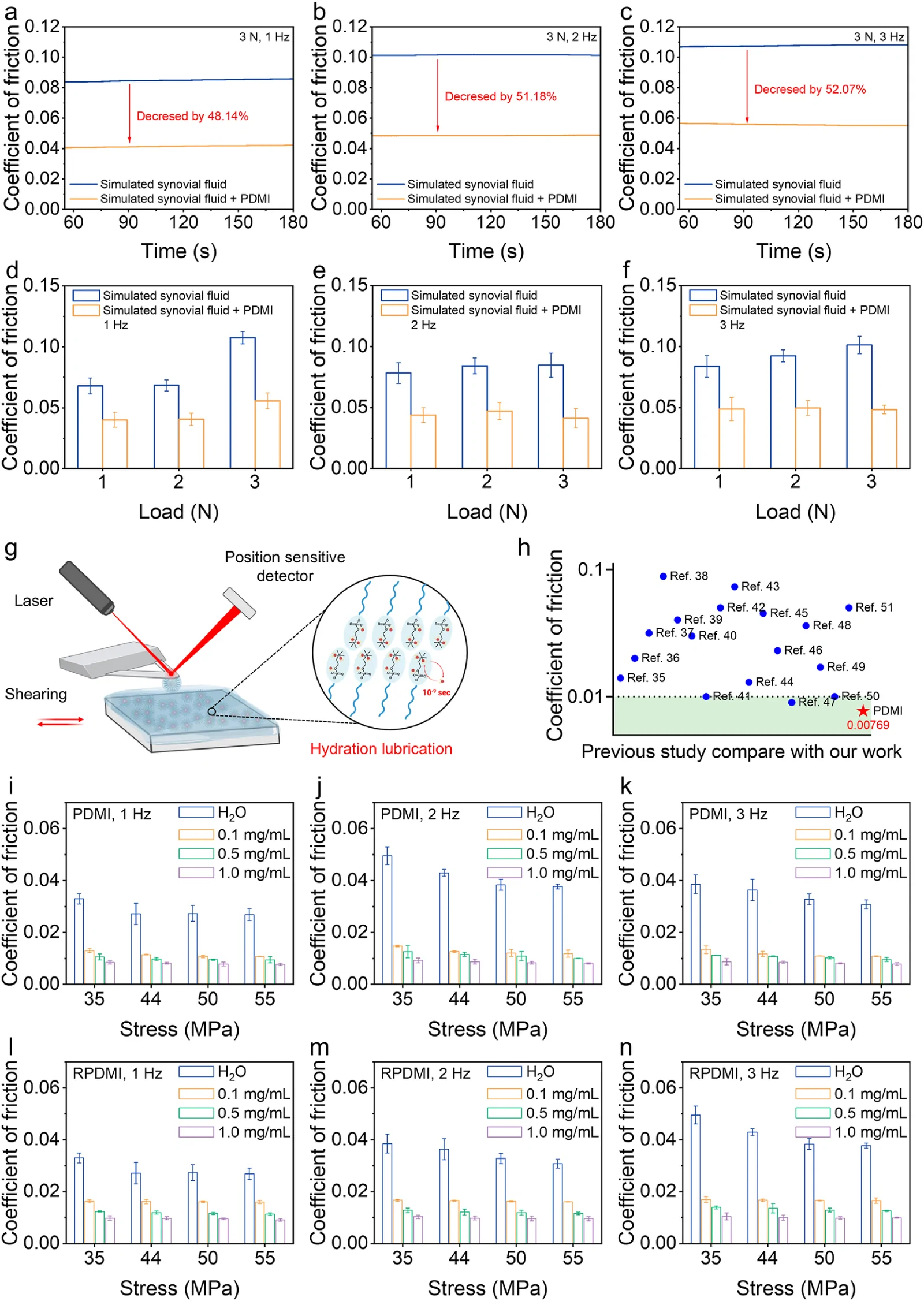
Lubrication performance characterization of PDMI and RPDMI. (a)-(c) Friction curves of simulated synovial fluid with or without PDMI under different sliding frequencies. (d)-(f) Quantitative comparison of the COF of simulated synovial fluid with or without PDMI. The data were presented as mean ± SD, n = 3. (g) Schematic diagram showing the experimental setup of nano-tribological test. (h) Comparison of COF values between our work and previous studies [[35], [36], [37], [38], [39], [40], [41], [42], [43], [44], [45], [46], [47], [48], [49], [50], [51]]. (i)-(k) Quantitative comparison of the COF of PDMI under different concentrations and frequencies tested by AFM. The data were presented as mean ± SD, n = 3. (l)-(n) Quantitative comparison of the COF of RPDMI under different concentrations and frequencies tested by AFM. The data were presented as mean ± SD, n = 3.

To evaluate the lubrication performance of PDMI under conditions more closely resembling the complex joint environment, the tribological test was conducted using UMT in simulated synovial fluid containing proteins and hyaluronic acid. The friction curves obtained at different loads and sliding frequencies were presented in Fig. 4a–c and Fig. S6, while the corresponding COF values at different loads and frequencies were compared in Fig. 4d–f and Fig. S7. In general, PDMI reduced the COF under all tested conditions, with the value decreasing from 0.1013 to 0.0485 at 3 N and 3 Hz, corresponding to a reduction of approximately 52.07%, thereby demonstrating stable lubrication under physiologically relevant conditions.

To further elucidate the lubrication behavior of PDMI at the microscale, atomic force microscopy (AFM)-based tribological tests were subsequently performed under different frequencies and loads, as shown in Fig. 4g. Compared with previous studies [[35], [36], [37], [38], [39], [40], [41], [42], [43], [44], [45], [46], [47], [48], [49], [50], [51]], the PDMI developed in this work exhibited a lower coefficient of friction (COF) in the level below 0.01 (Fig. 4h). Specifically, the PDMI exhibited superior lubrication compared with water across the entire frequency range (Fig. 4i–k). For example, at 1.0 mg/mL and 1 Hz, the COF of the PDMI was as low as 0.00769, whereas that of water under the same condition was 0.02686, which corresponded to a reduction of more than 70%. With the increase in the concentration of PDMI, the COF decreased monotonically. This behavior was attributed to the hydration lubrication mechanism, where the zwitterionic phosphorylcholine group in MPC strongly captured and orderly oriented free water molecules through electrostatic-dipole interaction [52,53], forming a hydration layer around an electrically neutral interface and thereby markedly reducing interfacial shear resistance [54,55]. PDMI did not rely on permanent adsorption onto the cartilage surface but remained dispersed as polymeric micelles and dynamically entered the contact region during joint motion. The continuous replenishment and reversible deformation of hydrated micelles within the confined interface further contributed to friction reduction. Furthermore, an increase in the frequency from 1 to 3 Hz led to a slight rise in COF (Fig. S8). However, at 1.0 mg/mL and 3 Hz, the PDMI still achieved a COF on the order of 0.001, indicating that a superlubricity regime was achieved under the tested conditions.

We further examined the lubrication behavior of RPDMI, the nanoparticles obtained after ROS-triggered drug release, as shown in Fig. 4l–n. After release, the COF increased slightly (Fig. S9), which might be due to the release of hydrophobic drugs weakening the stiffness of the PDMI, thereby reducing rolling lubrication. However, the COF of RPDMI still remained lower than that of pure water. This persistence arose because ROS cleavage removed only the drug side chains while preserving the polymer backbone. The remaining amphiphilic chains still self-assembled into stable nanoparticles and maintained the low-friction protection through the synergistic effects of hydration and rolling lubrication.

### 3.5 Biocompatibility, chondrocyte uptake, and immune evasion

**Fig. 5:**
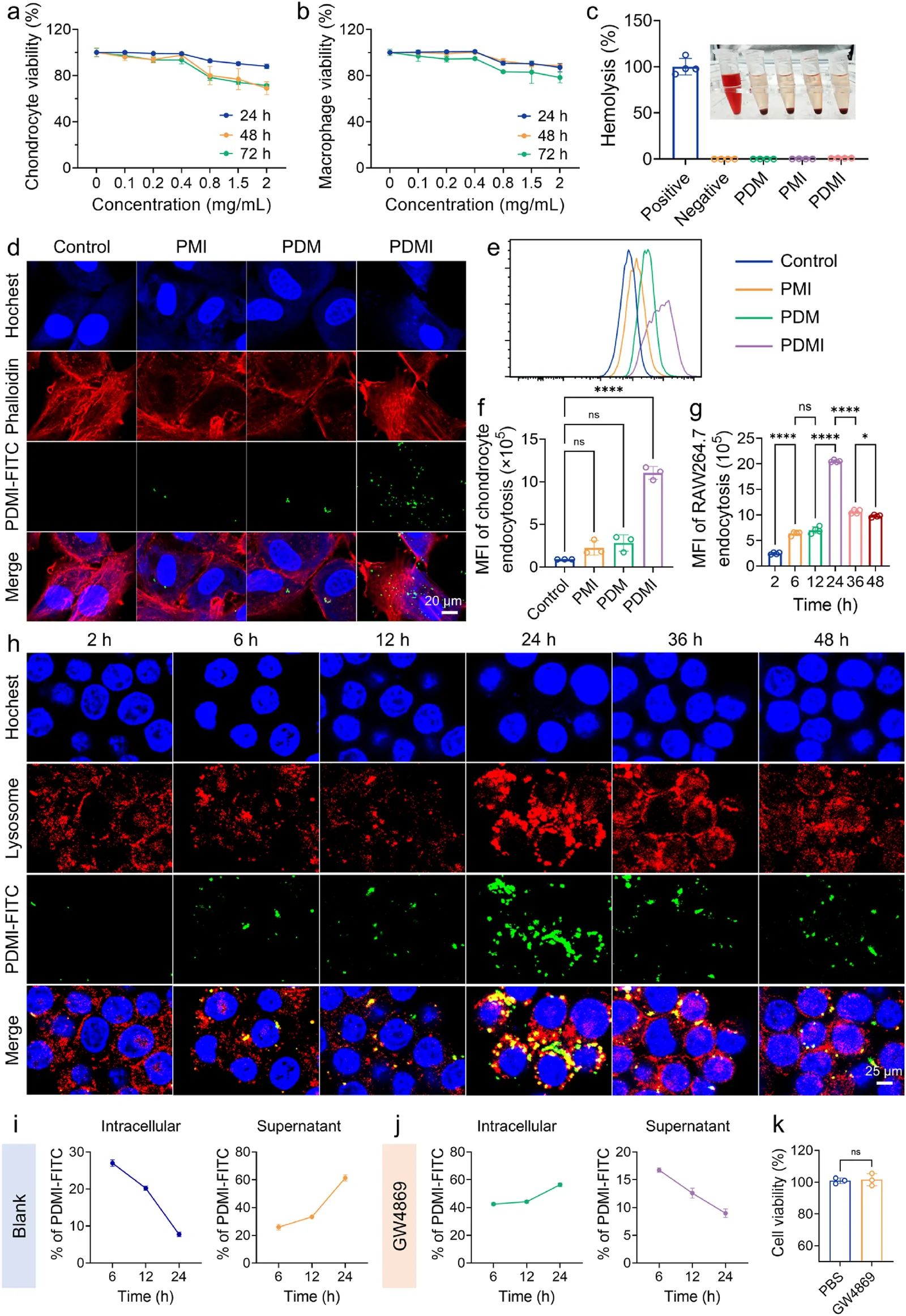
Biocompatibility, chondrocytes uptake, and immune escape characterization of PDMI. (a) Cell viability of chondrocytes cultured with PDMI at various concentrations. The data were presented as mean ± SD, n = 4. (b) Cell viability of macrophages incubated with PDMI at different concentrations. The data were presented as mean ± SD, n = 4. (c) Whole-blood hemolysis assay following incubation with PDMI. The data were presented as mean ± SD, n = 4. (d) Representative CLSM images of chondrocytes after 8 h incubation with PMI, PDM, and PDMI. FITC: fluorescein O-methacrylate. (e) FCM of chondrocytes cultured with PMI, PDM, and PDMI for 8 h. (f) Quantitative analysis of chondrocyte uptake for different groups detected by FCM. MFI: mean fluorescence intensity. The data were presented as mean ± SD, n = 3. ns indicated no significant difference (*p* ≥ 0.05), ****p < 0.0001. (g) Representative CLSM images of macrophages for immune escape assessment of PDMI after 48 h incubation. The data were presented as mean ± SD, n = 4. ns indicated no significant difference (*p* ≥ 0.05), *p < 0.05, ****p < 0.0001. (h) Quantitative analysis of macrophage uptake for different groups detected by FCM. (i) Time-dependent change in intracellular and supernatant FITC fluorescence intensities after PDMI internalization by RAW264.7. (j) Effect of the exocytosis inhibitor GW4869 on intracellular and supernatant FITC fluorescence intensities in RAW264.7. (k) Effect of GW4869 treatment on RAW264.7 viability. The data were presented as mean ± SD, n = 3. ns indicated no significant difference (p ≥ 0.05).

To evaluate the therapeutic potential of the PDMI for PTOA, cell counting kit-8 (CCK-8) assay was used to characterize cytotoxicity using primary rat chondrocytes and RAW264.7 macrophages (Fig. 5a–b). When the concentration of PDMI was below 400 μg/mL, both chondrocytes and macrophages maintained viability more than 98% even after 72 h of incubation. Because ICA was used as a bioactive modulator rather than a cytotoxic agent, the concentration of PDMI was selected as 400 μg/mL for subsequent *in vitro* experiments based on cytocompatibility rather than a conventional IC50 derived from cell growth inhibition. A rat whole-blood hemolysis assay further demonstrated that PMI (without dopamine), PDM, and PDMI all exhibited excellent biocompatibility, as shown in Fig. 5c. Live/dead staining (Fig. S10–S11) corroborated these findings, as no obvious dead cells were observed within the field of view, thereby indicating good biocompatibility.

Chondrocytes were the major cellular component of cartilage tissue. Accordingly, chondrocyte uptake was critical for regulating the intracellular milieu. Compared with the control group, both PDM and PDMI exhibited enhanced cellular internalization, which was partly associated with MPC-mediated interactions with the cell membrane (Fig. 5d). The higher uptake of PDMI than PDM was likely related to ICA conjugation-induced changes in particle size, surface potential, hydrophobicity, and polymer chain arrangement, which collectively promoted membrane contact and endocytosis. For flow cytometry (FCM) analysis, cellular debris and doublets were sequentially excluded using FSC-A/SSC-A and FSC-H/FSC-A gating, respectively, followed by quantification of the fluorescence intensity within the gated chondrocyte population (Fig. S12). The result further confirmed that PDMI exhibited the highest cellular internalization efficiency among all groups (Fig. 5e–f). Consequently, it indicated that PDMI could effectively enter the chondrocyte intracellular environment to modulate local oxidative stress. As key phagocytes of the immune system, macrophages typically mediated the rapid clearance of exogenous substances, leading to significant loss of efficacy. As MPC was a major constituent of cell membrane, it prevented the PDMI from being phagocytized by macrophage recognition, thereby conferring immune-evasive capability. Tracking PDMI in macrophages by CLSM and FCM showed that during the first 24 h, macrophages continuously internalized PDMI, with clear intensification of intracellular fluorescence (Fig. 5g–h). After 24 h, however, intracellular fluorescence decreased gradually. To verify that the detected fluorescence predominantly originated from PDMI-related nanostructures, PDMI was dual-labeled with FITC and maleimide-Cy5. The two fluorescence signals exhibited strong spatial colocalization, indicating that the major intracellular fluorescence remained associated with structurally retained PDMI rather than free dye or isolated degradation fragments (Fig. S13). After macrophage internalization, the intracellular fluorescence signal progressively decreased, whereas the fluorescence signal in the culture supernatant increased with time. The treatment with the exocytosis inhibitor GW4869 markedly reduced the supernatant fluorescence and enhanced intracellular retention without affecting cell viability (Fig. 5i–k), supporting an active vesicle-mediated extracellular release process rather than passive leakage caused by cell damage. TEM observation further revealed nanoparticle-like shape encapsulated within the membrane-bound vesicle-like structure isolated from the culture supernatant of RAW264.7 after 48 h of incubation, and the morphology and electron density of the encapsulated nanoparticle were consistent with those of PDMI (Fig. S14). This evidence further supported that internalized PDMI was released into the extracellular environment through vesicle-mediated exocytosis, consistent with the CLSM and FCM results. Macrophage-mediated clearance of nanomaterials is influenced by the interaction between complement C3 deposited on nanoparticle surfaces and the macrophage receptor CD11b. Previous studies have shown that PMPC-mediated hydration layer could reduce complement C3 deposition and attenuate C3/CD11b-mediated macrophage recognition [23], while our C3 opsonization experiment (Fig. S15) further demonstrated that inhibiting C3 deposition on PDMI decreased macrophage uptake, supporting the involvement of complement-mediated recognition in its immune clearance. These findings suggested that the zwitterionic MPC interface may contribute to immune evasion by reducing complement-related opsonization and subsequent macrophage uptake.

### 3.6 Biological mechanism of chondrocyte regulation

**Fig. 6:**
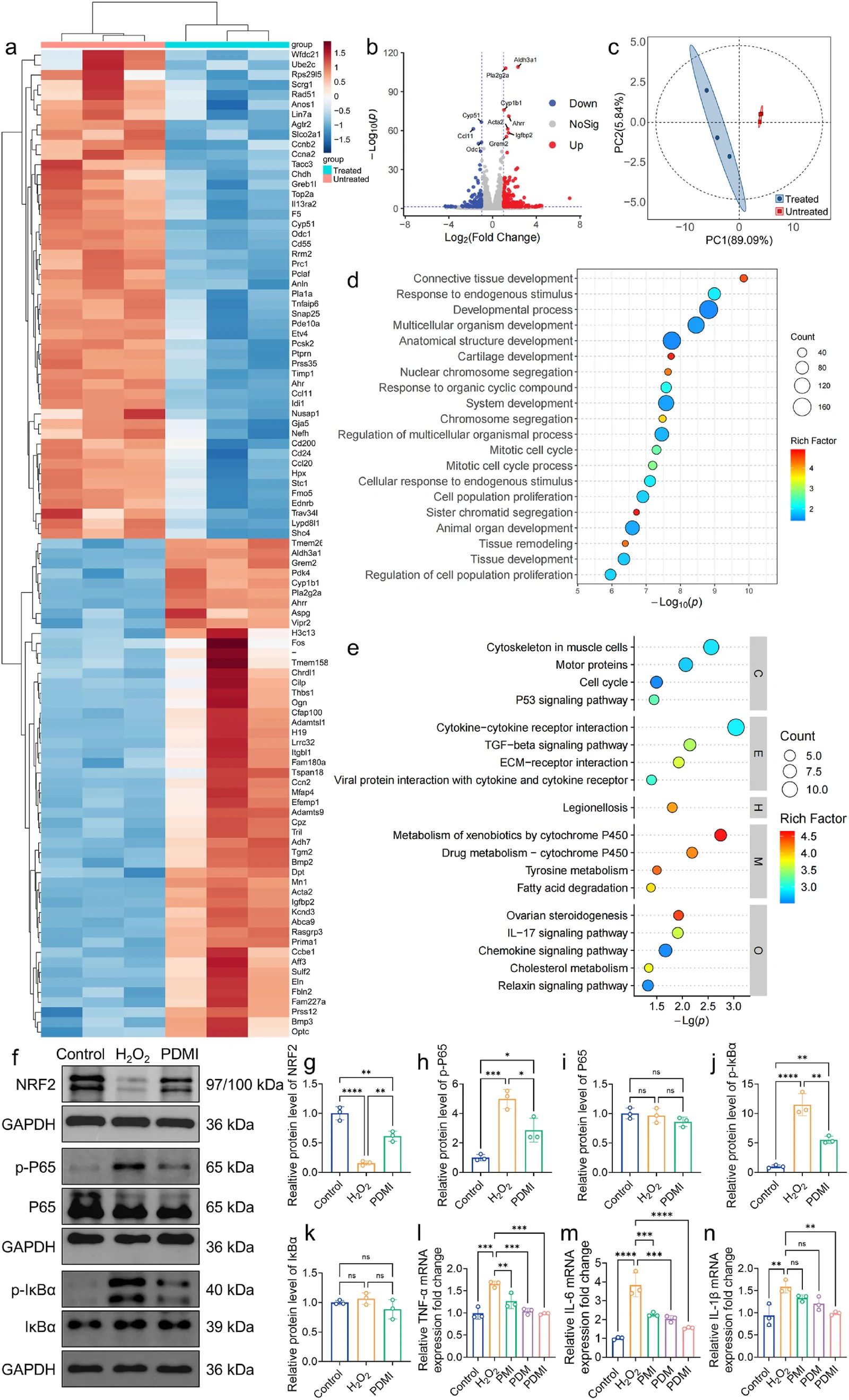
Biological mechanism of PDMI exerting anti-inflammatory effects. (a) Heatmap of DEGs. (b) Volcano plot. (c) PCA plot. (d) GO enrichment analysis of DEGs. (e) KEGG enrichment analysis of DEGs. (f) Western blot analysis of antioxidant and anti-inflammatory signaling pathway-related proteins in chondrocytes. (g)-(k) Quantitative analysis of the relative protein expression levels of NRF2, p-P65, P65, p-IκBα, and IκBα in chondrocytes, respectively. All protein levels were normalized to GAPDH. The data were presented as mean ± SD, n = 3. ns indicated no significant difference (*p* ≥ 0.05), *p < 0.05, **p < 0.01, ***p < 0.001, ****p < 0.0001. (l)-(n) RT-qPCR analysis of the relative mRNA expression levels of TNF-α, IL-6, and IL-1β in chondrocytes subjected to different treatments, respectively. The data were presented as mean ± SD, n = 3. ns indicated no significant difference (*p* ≥ 0.05), **p < 0.01, ***p < 0.001, ****p < 0.0001.

To investigate the effect of PDMI on regulation of chondrocyte function, RNA sequencing was performed on lipopolysaccharide (LPS)-treated chondrocytes, which were co-cultured with the PDMI, and differentially expressed genes (DEGs) were analyzed. LPS was used as a defined inflammatory stimulus to establish a reproducible *in vitro* model for evaluating inflammation-related transcriptional responses in chondrocytes. The heatmap (Fig. 6a) indicated that, compared with the LPS group, the PDMI downregulated key inflammation and mitochondrial oxidative stress-related genes (e.g., Ccl20, Chdh, Il13ra2) while upregulating key genes associated with metabolism and ECM synthesis (e.g., Bmp2, Efemp1, Itgbl1). The volcano plot shown in Fig. 6b and minus-versus-add (MA) plot displayed in Fig. S16 summarized the overall DEG distribution, identifying a total of 218 significantly upregulated and 156 significantly downregulated genes. Notably, the hallmark inflammatory genes such as Ccl11 and Odc1 were markedly reduced after treatment with the PDMI. Principal component analysis (PCA, Fig. 6c) showed significant separation between LPS group and PDMI group, indicating complete transcriptional changes.

Furthermore, Gene Ontology (GO) enrichment analysis (Fig. 6d) demonstrated enrichment of processes highly related to connective tissue development and inflammatory or stress responses, whereas Kyoto Encyclopedia of Genes and Genomes (KEGG) analysis (Fig. 6e) highlighted pathways linked to ECM synthesis and inflammatory metabolism, including TGF-beta signaling pathway and ECM-receptor interaction. A Sankey diagram shown in Fig. S17 connected DEGs to multiple enriched pathways and emphasized central genes affecting both inflammation and mitochondrial function.

Western blot analysis further validated these molecular changes (Fig. 6f–k). The oxidative stress stimulation reduced NRF2 expression to approximately 15.86% of the control level and increased the p-P65/P65 and p-IκBα/IκBα ratios by approximately 5.16-fold and 10.76-fold, respectively. PDMI treatment restored NRF2 expression to approximately 61.27% of the control level and reduced the two phosphorylation ratios by approximately 35.47% and 42.04%, respectively, indicating activation of NRF2-mediated antioxidant defense and suppression of the NF-κB/IκBα signaling pathway. RT-qPCR analysis further corroborated the anti-inflammatory effect of PDMI at the transcriptional level (Fig. 6l–n). Compared with the control group, oxidative stress stimulation increased the mRNA expression levels of TNF-α, IL-6, and IL-1β by approximately 1.67-fold, 3.83-fold, and 1.69-fold, respectively. PDMI treatment reduced the expression levels of these inflammatory cytokines by approximately 40.43%, 59.54%, and 37.71%, respectively, further confirming that PDMI suppressed downstream inflammatory responses associated with NF-κB/IκBα pathway activation. Collectively, the Western blot and RT-qPCR results further supported that PDMI exerted antioxidant and anti-inflammatory effects by restoring NRF2-mediated antioxidant defense and suppressing NF-κB/IκBα pathway activation and downstream inflammatory cytokine expression.

### 3.7 Mitochondrial protection and cartilage matrix restoration

**Fig. 7:**
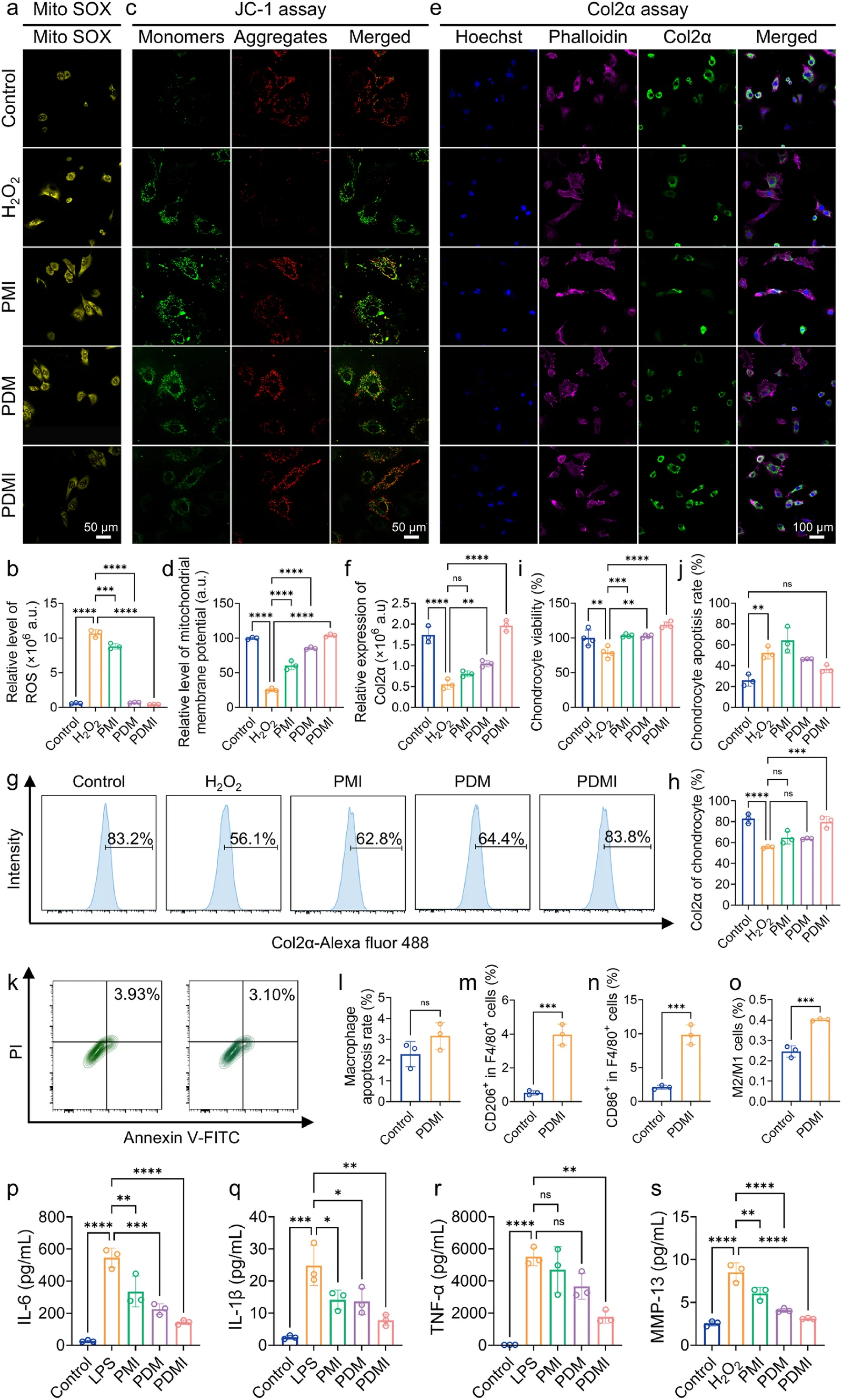
Characterization of PDMI in function regulation of inflammatory microenvironment, protecting chondrocytes and anti-inflammation. (a) Representative fluorescence images of mitochondrial superoxide levels in chondrocytes detected using the MitoSOX assay. (b) Quantitative analysis of the relative MitoSOX fluorescence intensity. The data were presented as mean ± SD, n = 3. ***p < 0.001, ****p < 0.0001. (c) Representative fluorescence images of mitochondrial membrane potential detected using the JC-1 assay, with JC-1 monomers shown in green and JC-1 aggregates shown in red. (d) Quantitative analysis of mitochondrial membrane potential based on the red-to-green fluorescence intensity ratio. The data were presented as mean ± SD, n = 3. ****p < 0.0001. (e) Representative immunofluorescence images of Col2α expression in chondrocytes, with nuclei stained by Hoechst in blue, F-actin stained by phalloidin in magenta, and Col2α shown in green. (f) Quantitative analysis of the relative Col2α fluorescence intensity. The data were presented as mean ± SD, n = 3. ns indicated no significant difference (*p* ≥ 0.05), **p < 0.01, ****p < 0.0001. (g) Representative FCM plots of Col2α expression level in chondrocytes. (h) Statistical analysis of Col2α expression level in chondrocytes. The data were presented as mean ± SD, n = 3. ns indicated no significant difference (*p* ≥ 0.05), ***p < 0.001, ****p < 0.0001. (i) CCK-8 assay of the viability of inflammatory chondrocytes treated with PDMI. The data were presented as mean ± SD, n = 4. **p < 0.01, ***p < 0.001, ****p < 0.0001. (j) Inflammatory chondrocytes apoptosis rate. The data were presented as mean ± SD, n = 3. ns indicated no significant difference (*p* ≥ 0.05), **p < 0.01. (k) FCM analysis of macrophage viability after incubation with PDMI. (l) Macrophage apoptosis rate obtained by flow cytometry after co-culturing with PDMI. The data were presented as mean ± SD, n = 3. ns indicated no significant difference (*p* ≥ 0.05). (m) M2 phenotype polarization rate of macrophages. The data were presented as mean ± SD, n = 3. ***p < 0.001. (n) M1 phenotype polarization rate of macrophages. The data were presented as mean ± SD, n = 3. ***p < 0.001. (o) Ratio of M2 phenotype to M1 phenotype in macrophages. The data were presented as mean ± SD, n = 3. ***p < 0.001. (p)-(r) Expression levels of IL-6, IL-1β, and TNF-α collected for macrophages. The data were presented as mean ± SD, n = 3. ns indicated no significant difference (*p* ≥ 0.05), *p < 0.05, **p < 0.01, ***p < 0.001, ****p < 0.0001. (s) Expression level of MMP-13 collected for chondrocytes. The data were presented as mean ± SD, n = 3. **p < 0.01, ****p < 0.0001.

In PTOA progression, the imbalance of redox homeostasis and excessive intracellular generation of ROS in the microenvironment were recognized as key drivers [56,57]. Under inflammatory conditions, mitochondrial homeostasis was disrupted, resulting in impaired energy supply, dysregulated mitochondrial calcium uptake, and accelerated chondrocyte apoptosis. Consequently, scavenging excessive ROS was very critical for preserving mitochondrial integrity and reducing chondrocyte apoptosis. To evaluate mitochondrial ROS levels in chondrocytes, we utilized the mitochondria-specific ROS probe MitoSOX, followed by fluorescence imaging and quantification (Fig. 7a–b). The result showed a slight decrease in fluorescence intensity after treatment with PMI or PDM. However, chondrocytes treated with PDMI exhibited a pronounced reduction in fluorescence signal, indicating superior efficacy in scavenging mitochondrial ROS. In addition, mitochondrial membrane potential disruption was an early hallmark of cell apoptosis. Under physiological condition, mitochondria maintained a relatively high membrane potential, allowing JC-1 dye to accumulate within the mitochondrial matrix and generate red fluorescence. Upon mitochondrial injury, JC-1 failed to aggregate and remained as monomers instead, thus producing green fluorescence. Chondrocytes exposed to H_2_O_2_ displayed strong green fluorescence, indicating that inflammation stimulated mitochondrial damage and reduced membrane potential (Fig. 7c–d). PMI or PDM treatment did not cause marked changes, whereas PDMI-treated chondrocytes displayed stronger red fluorescence with attenuated green signals. The result indicated that PDMI provided significant protection against oxidative stress.

During PTOA progression, inflammatory mediators and excessive ROS jointly led to ECM degradation. Col2α, a major component of cartilage ECM, was markedly downregulated under inflammatory conditions [58]. Col2α expression was evaluated by immunofluorescence staining, and PDMI treatment significantly restored Col2α expression, as shown in Fig. 7e–f. FCM further quantitatively confirmed that PDMI significantly attenuated Col2α degradation (Fig. 7g–h), indicating that PDMI had potential in restoring ECM homeostasis.

### 3.8 Chondrocyte protection and anti-inflammatory activity

To further evaluate the protective effect of PDMI under pathological conditions, chondrocytes were exposed to H_2_O_2_ to simulate the oxidative inflammatory microenvironment and subsequently subjected to cell viability and apoptosis assays. PDMI efficiently preserved chondrocyte viability even higher than control group (Fig. 7i). Besides, to distinguish the contribution of ROS-responsive ICA release from those of free ICA and the carrier, chondrocyte viability under H_2_O_2_-induced oxidative stress was compared among free ICA, PDM plus ICA, and PDMI. As shown in Fig. S18, PDMI significantly improved chondrocyte viability compared with free ICA and maintained cell viability at a level comparable to that of the control group. In contrast, the combination of PDM and ICA showed no significant difference from free ICA under the same condition. These results indicated that ROS-responsive ICA delivery played an important role in the chondroprotective effect of PDMI rather than the effect arising solely from PDM or freely added ICA. FCM analysis further revealed that PDMI significantly inhibited chondrocyte apoptosis under inflammatory condition (Fig. 7j). In addition, the result in Fig. 7k–l indicated that PDMI did not achieve anti-inflammatory effect by inducing macrophage apoptosis. For macrophage polarization analysis, F4/80-positive macrophages were first gated, followed by quantification of the CD86-positive and CD206-positive populations representing the M1 and M2 phenotypes, respectively (Fig. S19). The representative FCM plot and quantitative result showed that PDMI markedly promoted macrophage polarization toward the M2 phenotype (Fig. 7m–o), indicating that its anti-inflammatory effect was primarily mediated through macrophage reprogramming rather than apoptosis.

Furthermore, in PTOA progression, activated macrophages proliferated and secreted inflammatory mediators, ultimately leading to vasodilation, tissue edema, and cartilage destruction. The expression levels of inflammatory factors such as interleukin-6 (IL-6), IL-1β, and TNF-α were examined to evaluate the anti-inflammatory function of the PDMI using enzyme-linked immunosorbent assay (ELISA). As shown in Fig. 7p–r, LPS stimulation increased TNF-α, IL-1β, and IL-6 production in macrophages. Compared with the LPS group, both PMI and PDM reduced cytokine levels, whereas PDMI treatment further suppressed cytokine production to the level similar to control group, highlighting its potent anti-inflammatory activity and its ability to modulate the PTOA microenvironment. In addition to oxidative stress, we further characterized the expression level of MMP-13, a critical enzyme implicated in PTOA progression that degraded ECM components and contributed to joint pain. As shown in Fig. 7s, PDMI treatment significantly suppressed MMP-13 expression level, demonstrating its protective effect against cartilage degradation.

### 3.9 Intra-articular retention evaluation

**Fig. 8:**
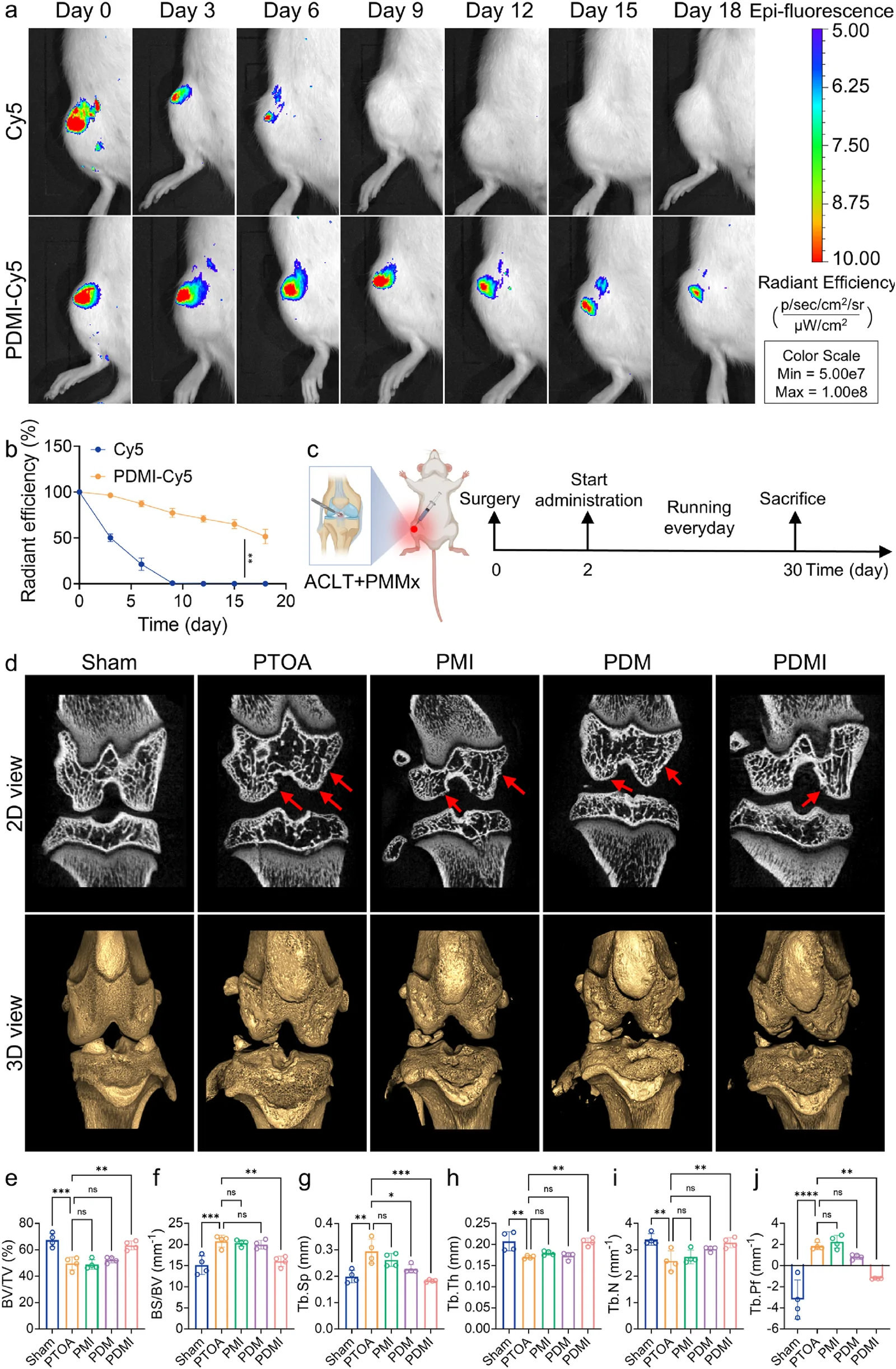
Characterization of *in vivo* retention and therapeutic effect of the PDMI for PTOA treatment. (a) Representative fluorescence images illustrating intra-articular retention of PDMI. (b) Statistical analysis of radiant efficiency of intra-articular retention of PDMI. (c) Establishment of PTOA model and treatment timeline for intra-articular injection and postoperative management. (d) Representative 2D micro-CT and 3D reconstruction images after 4 weeks of treatment for different groups. (e-i) Quantification of microstructural parameters of subchondral bone, including BV/TV, BS/BV, Tb.Sp, Tb.Th, and Tb.N, respectively. The data were presented as mean ± SD, n = 4. ns indicated no significant difference (*p* ≥ 0.05), *p < 0.05, **p < 0.01, ***p < 0.001, ****p < 0.0001.

To evaluate the *in vivo* intra-articular retention of the PDMI, Cy5-labeled PDMI was injected into the rat knee joint, and its distribution was monitored using an *in vivo* imaging system for 21 consecutive days. As shown in Fig. 8a–b, the fluorescence of free Cy5 disappeared completely by 9 days post-injection, whereas PDMI-Cy5 retained more than 50% of its fluorescence intensity 18 days after intra-articular administration. One-phase exponential decay fitting (Fig. S20) showed that the Cy5-labeled PDMI exhibited an apparent intra-articular retention half-life of 22.93 days, which was approximately 8.9-fold longer than that of free Cy5 at 2.58 days.

### 3.10 Therapeutic efficacy of PDMI

To assess the therapeutic potential of PDMI *in vivo*, we employed a rat PTOA model by performing anterior cruciate ligament transection (ACLT) combined with partial medial meniscectomy (pMMx) on the right knee (Fig. 8c). Two days post-surgery, the rats were subjected to daily running training and a single intra-articular injection of the PDMI to evaluate the therapeutic efficacy. Four weeks after treatment, micro-computed tomography (micro-CT) was performed to characterize osteophyte formation and subchondral bone remodeling. Representative 2D micro-CT slices and 3D reconstructions revealed increased bone-surface roughness and osteophyte formation in the PTOA group, which were characteristic manifestations of abnormal subchondral bone remodeling during PTOA progression (Fig. 8d). However, PDMI exhibited a smoother bone surface morphology compared with PTOA group. Quantitative analysis of subchondral bone microarchitecture, including bone volume fraction (BV/TV), bone surface to bone volume ratio (BS/BV), trabecular separation (Tb.Sp), trabecular thickness (Tb.Th), and trabecular number (Tb.N), further confirmed the therapeutic effects (Fig. 8e–i). BV/TV reflected relative bone mass, BS/BV indicated bone-surface complexity, while Tb.N, Tb.Sp, and Tb.Th represented trabecular number, spacing, and thickness, respectively. Abnormal changes in these parameters indicated disruption of the subchondral bone microarchitecture. Following PDMI treatment, these parameters shifted toward the values observed in the Sham group and differed significantly from those in the PTOA group. These findings indicated that PDMI attenuated osteophyte formation and abnormal subchondral bone remodeling during the 4-week observation period.

### 3.11 Histological evaluation

**Fig. 9:**
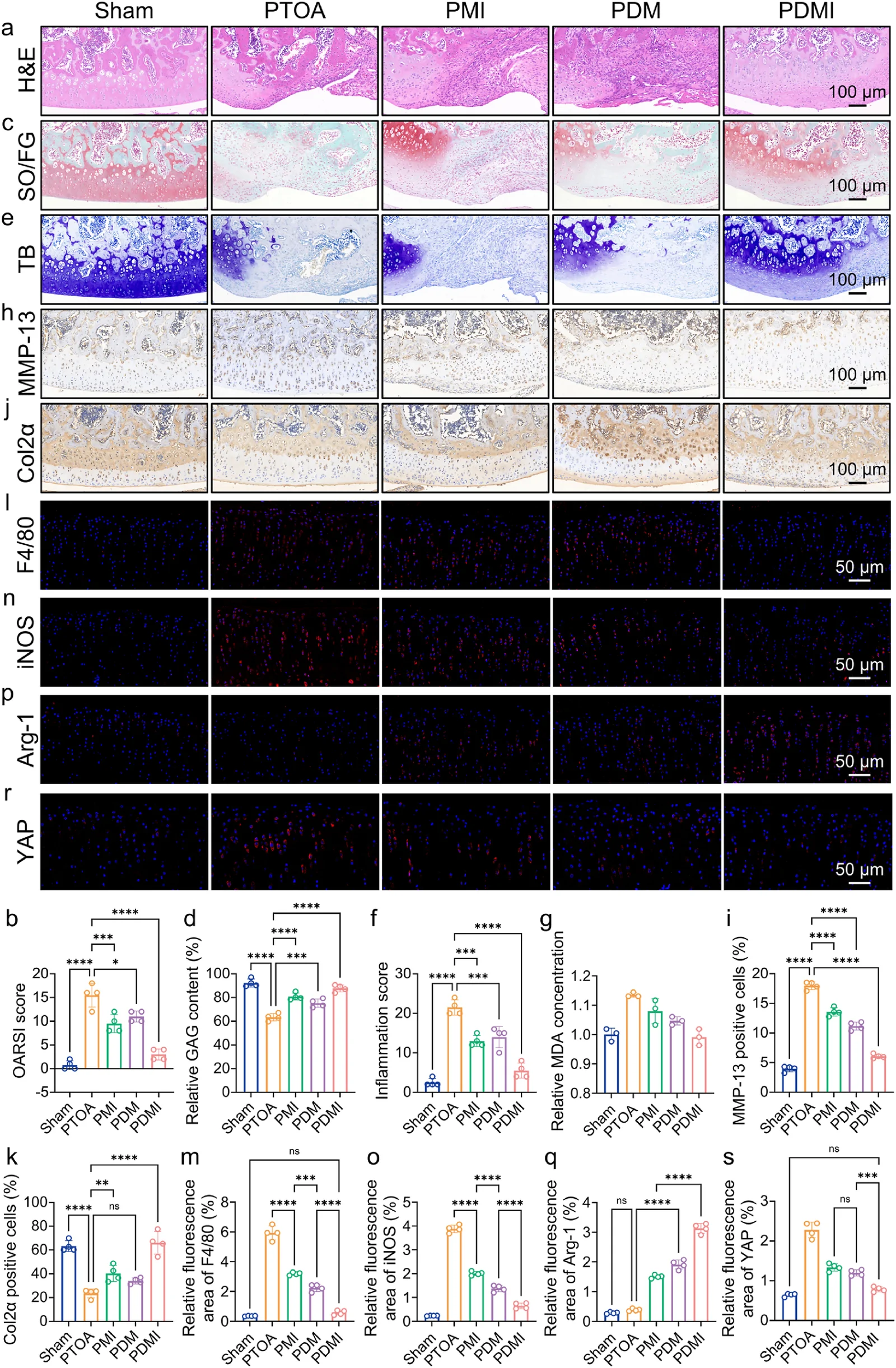
Histological, immunohistochemical, and immunofluorescence evaluation of articular cartilage degeneration, extracellular matrix homeostasis, and macrophage responses in rat knee joints after 4 weeks of treatment. Representative images of (a) hematoxylin and eosin staining, (c) safranin O/fast green staining, and (e) toluidine blue staining. Quantitative analyses of (b) OARSI scores, (d) relative glycosaminoglycan content, and (f) inflammation scores. The data were presented as mean ± SD, n = 4. *p < 0.05, ***p < 0.001, ****p < 0.0001. Representative immunohistochemical images of (g) MDA levels in articular cartilage tissues. The data were presented as mean ± SD, n = 3. (h) MMP-13 and (j) Col2α expression. Quantitative analyses of (i) MMP-13-positive cells and (k) Col2α-positive cells. The data were presented as mean ± SD, n = 4. ns indicated no significant difference (*p* ≥ 0.05), **p < 0.01, ****p < 0.0001. Representative immunofluorescence images of (l) F4/80, (n) iNOS, (p) Arg-1 and (r) YAP expression. Quantitative analyses of (m) F4/80, (o) iNOS, (q) Arg-1 and (s) YAP expression. The data were presented as mean ± SD, n = 4. ns indicated no significant difference (p ≥ 0.05), ***p < 0.001, ****p < 0.0001.

To characterize cartilage structural changes, histological analyses were performed using hematoxylin and eosin (H&E), safranin O/fast green (SO/FG), and toluidine blue (TB) staining. H&E staining revealed that cartilage in the sham group displayed a continuous and intact tangential layer, with evenly distributed cytoplasmic and matrix components, and orderly longitudinal arrangement of chondrocytes in transitional zone. Conversely, the samples in PTOA group exhibited distinct pathological features including thinning and disorganization of the tangential layer, surface destruction of the perichondrium, extrusion and clustering of chondrocytes, disordered arrangement of chondrocytes in transitional zone, localized chondrocyte calcification, and disrupted tidemark adhesion between calcified cartilage and subchondral bone. Notably, the PDMI treatment group showed significant improvement, with cartilage tissue morphology resembling that of the sham group (Fig. 9a). Furthermore, PDMI reduced Osteoarthritis Research Society International (OARSI) score by more than 80% compared with the PTOA group (Fig. 9b). Additionally, we evaluated ECM changes using SO/FG staining. As shown in Fig. 9c, the PTOA group exhibited markedly diminished staining intensity relative to the sham group, indicating glycosaminoglycan (GAG) loss and exacerbated cartilage wear. By contrast, the staining intensity progressively recovered after treatment with PMI, PDM, and PDMI, indicating improved ECM continuity, restoration of cartilage architecture, and fewer fissures. Notably, the treatment with PDMI produced a relative GAG content comparable with the sham group (Fig. 9d). Furthermore, TB staining supported the cartilage-protective effect of PDMI. Following PDMI treatment, the cartilage surface appeared smoother and the ECM distribution was greatly improved (Fig. 9e). Inflammation score in Fig. 9f showed that, compared with the PTOA group, PMI and PDM reduced inflammation by 39.53% and 34.88%, respectively, primarily reflecting the active regulation by ICA in PMI and passive protective effect of DMA in PDM. Combining these two mechanisms, PDMI reduced the inflammation score by 74.42%, further supporting its anti-inflammatory effect through active-passive synergistic regulation. Consistent with these findings, the MDA levels in articular cartilage tissues were markedly elevated in the PTOA group and decreased after PMI, PDM, and PDMI treatment, with PDMI producing the greatest reduction and restoring MDA levels close to those of the sham group (Fig. 9g). This result further demonstrated that PDMI attenuated lipid peroxidation and oxidative damage *in vivo*.

### 3.12 Immunohistochemical and immunofluorescence analyses

To further assess therapeutic efficacy, we performed immunohistochemical staining to examine MMP-13 and Col2α expression levels in cartilage tissue, thereby evaluating the pathological status of articular cartilage. As shown in Fig. 9h–k, the PTOA group exhibited significantly increased MMP-13 and markedly reduced Col2α compared with the sham group, which was consistent with matrix degradation and aggravated cartilage erosion. Following PMI or PDM treatment, MMP-13 expression decreased (Fig. 9i) while Col2α increased in a certain degree (Fig. 9k), indicating effective attenuation of cartilage degeneration. Notably, PDMI produced the most pronounced regulation of both markers, reducing MMP-13 and elevating Col2α expression to levels approaching those of the sham group. The result further highlighted the significant role of PDMI in protecting cartilage and mitigating degeneration. Together with SO/FG and TB staining, Col2α immunofluorescence, flow cytometric analysis, and the molecular pathway result, these findings provided multilevel evidence that PDMI attenuated cartilage matrix degradation and maintained ECM homeostasis at the tissue, cellular, and molecular levels.

To further evaluate macrophage responses within the joint microenvironment, immunofluorescence staining for F4/80, iNOS, and Arg-1 was performed (Fig. 9l, n, and 9p). Compared with the Sham group, the PTOA group exhibited markedly increased F4/80 and iNOS fluorescence, by approximately 16.65-fold and 16.59-fold, respectively (Fig. 9m and o), whereas Arg-1 expression remained relatively low (Fig. 9q), indicating enhanced macrophage infiltration and predominant pro-inflammatory activation. PDMI treatment reduced the relative fluorescence areas of F4/80 and iNOS by approximately 89.64% and 51.34%, respectively, while increasing Arg-1 fluorescence by approximately 8.10-fold compared with the PTOA group. These findings demonstrated that PDMI alleviated inflammatory macrophage infiltration and promoted macrophage transition toward an anti-inflammatory and reparative phenotype *in vivo*, consistent with the *in vitro* macrophage polarization results.

In addition, YAP immunofluorescence staining was performed to evaluate the mechanotransduction-related responses in articular cartilage (Fig. 9r–s). Compared with the sham group, the PTOA group showed markedly increased YAP fluorescence intensity by approximately 2.53-fold, indicating abnormal activation of mechanosensitive signaling after joint injury. PDMI treatment reduced YAP expression by approximately 65.47% compared with the PTOA group, and this inhibitory effect was more pronounced than that observed in the PMI and PDM groups, indicating that PDMI alleviated aberrant mechanotransduction activation *in vivo*. This result was consistent with the superior lubrication performance of PDMI and indicated that PDMI could contribute to cartilage protection by improving the mechanical microenvironment of the joint.

### 3.13 Biosafety evaluation

**Fig. 10:**
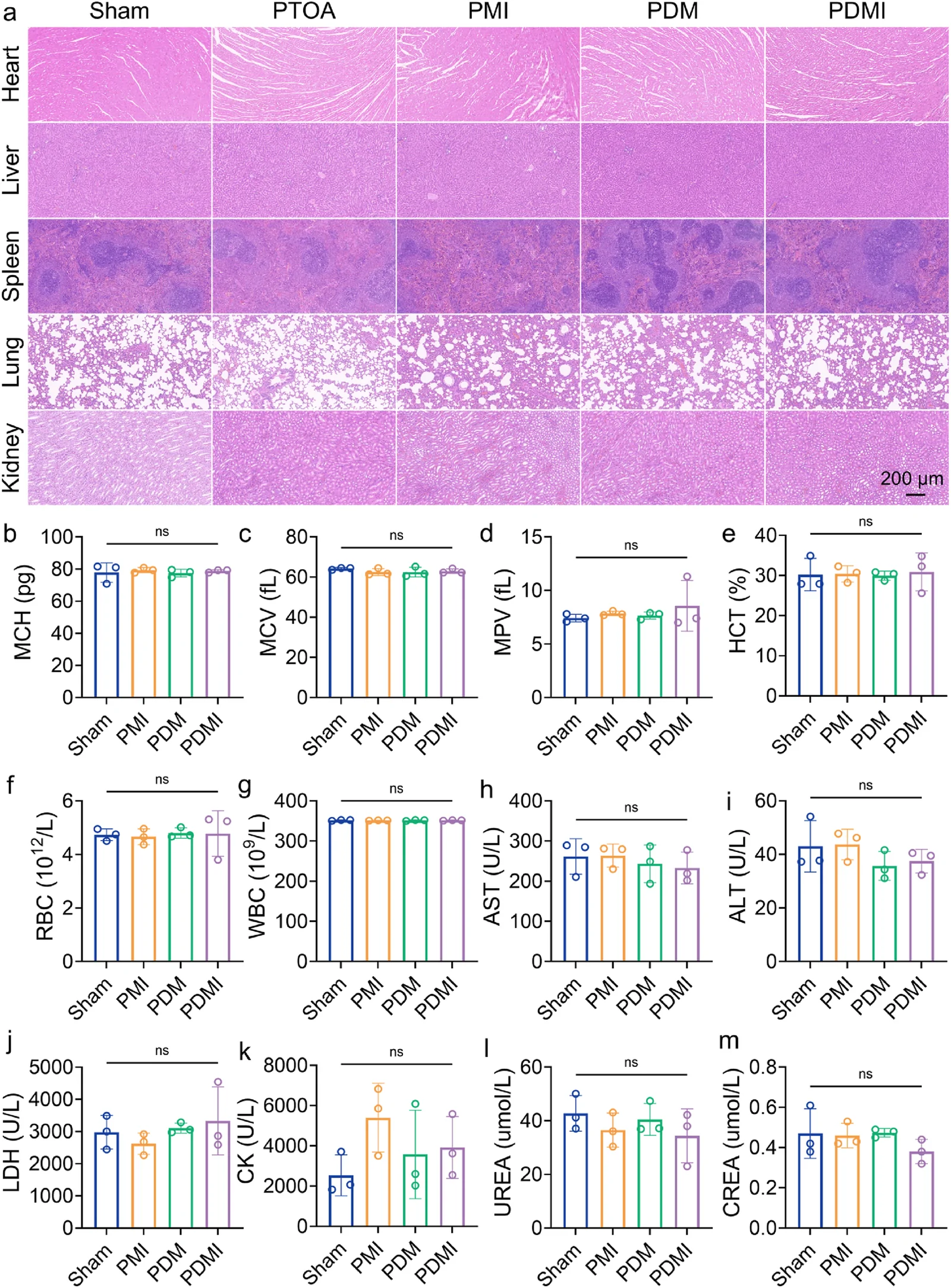
Evaluation of *in vivo* systemic toxicity. (a) Biocompatibility characterization of the PDMI by H&E staining of rat organs including heart, liver, spleen, lung, and kidney. (b-g) Biosafety characterization based on complete blood count, including (b) mean corpuscular hemoglobin (MCH), (c) mean corpuscular volume (MCV), (d) mean platelet volume (MPV), (e) red blood cell count (RBC), (f) white blood cell count (WBC), and (g) hematocrit (HCT). The data were presented as mean ± SD, n = 3. ns indicated no significant difference (*p* ≥ 0.05). (h-m) Biosafety characterization based on serum biochemical analysis, including (h) alanine aminotransferase (ALT), (i) aspartate aminotransferase (AST), (j) lactate dehydrogenase (LDH), (k) creatine kinase (CK), (l) blood urea nitrogen (UREA), and (m) creatinine (CREA). The data were presented as mean ± SD, n = 3. ns indicated no significant difference (*p* ≥ 0.05).

Biosafety is a critical consideration for evaluating clinical applicability of biomaterials. To investigate the potential toxicity of PDMI, the major organs including heart, liver, spleen, lung, and kidney were collected after 4 weeks of treatment and subjected to histopathological examination based on H&E staining. As shown in Fig. 10a, no pathological abnormalities such as inflammation, edema, or necrosis were observed in any organs from sham, PTOA, PMI, PDM, or PDMI group, indicating that the injection of the materials did not induce systemic toxicity or organ damage during metabolism. Additionally, the complete blood count (Fig. 10b–g) and serum biochemical analysis (Fig. 10h–m) further confirmed the absence of systemic toxicity. Collectively, these results jointly indicated that the PDMI -mediated intra-articular therapy for PTOA possessed excellent *in vivo* biocompatibility and biosafety.

## 4 Discussion

As society develops and living standards rise, more young people engage in fitness and sports. However, suboptimal training practices can increase the risk of joint mechanical injury. Unlike degradative OA caused by interfacial mechanical wear, PTOA affects the individuals in all age groups. Sprains, sports strains, and fractures all precipitate PTOA and are particularly common among the athletes and military personnel. Because PTOA has a prolonged latency, its severe consequences are often overlooked. By the time pain is perceived, pathology has typically advanced, and total joint replacement may be the only recourse to treat this disease. The developed PDMI system was evaluated for early intervention in PTOA and produced significant cartilage-protective effects during the 4-week observation period, supporting its potential as an intra-articular therapeutic strategy for early-stage PTOA.

The core innovation lay in the synergy of a multilayered defense-and-intervention strategy based on “Trojan horse” bioinspiration. Specifically, MPC-mediated immune escape and hydration lubrication formed an outer physical barrier that greatly reduced immune system recognition and interfacial shear, the catechol structure of DMA acted as the first antioxidative barrier, preferentially eliminating excessive ROS, and finally the phenylboronate ester served as a second, chemically responsive unit that cleaved under high ROS levels to release ICA on demand, enabling a seamless transition from passive scavenging to active intervention. Converging the *in vitro* and *in vivo* evidence supported the above mechanism. AFM testing yielded COF values on the order of 0.001, indicating a superlubricity regime under the tested microscale conditions, while transcriptomic analysis revealed downregulation of inflammation- and mitochondrial oxidative stress-related pathways. Mitochondrial probe and membrane-potential assays demonstrated significant reduction in mitochondrial ROS and stabilization of membrane potential. In addition, macrophage tracking showed early phagocytosis followed by exocytosis-mediated immune escape and induction of M2 polarization to reprogram the microenvironment. Furthermore, intra-articular imaging exhibited >50% fluorescence retention even 18 days after a single injection. Together with the histological and immunohistochemical findings, including reduced OARSI scores, restored GAG content, decreased MMP-13 expression, and increased Col2α expression, these results supported the short-term protective effect of PDMI on cartilage homeostasis during the early stage of PTOA. Collectively, these findings suggested that PDMI reduced friction-related mechanical stimulation at the contact interface and alleviated inflammation- and oxidative stress-associated ECM degradation through enhanced lubrication, ROS scavenging, ROS-responsive ICA release, and regulation of the inflammatory microenvironment.

From a translational perspective, a single intra-articular administration resulted in prolonged local retention, reduced inflammatory responses, and improved cartilage histological features during the observation period, thereby partially addressing the rapid clearance associated with conventional intra-articular treatments. Nevertheless, the present study primarily evaluated the early effect and short-term safety of a single intra-articular administration of PDMI in a 4-week accelerated PTOA model. Future studies will employ 12- and 24-week models and repeated-dose regimens to assess sustained efficacy, local and systemic toxicity, potential immunogenicity, cumulative effect, functional recovery, and interaction with commonly used drugs or other intra-articular therapies. In parallel, the durability of the lubrication effect will be further examined using cartilage-to-cartilage contact models under physiologically relevant loading and cyclic motion conditions. Although the present immunohistochemical, immunofluorescence, histological, flow cytometric, and molecular analyses collectively supported the regulation of ECM homeostasis by PDMI, direct quantitative validation of key matrix-related proteins remained limited. Future studies will employ western blotting and proteomic analysis to quantify MMP-13, Col2α, and other ECM-associated proteins and to more systematically elucidate the effects of PDMI on cartilage matrix synthesis and degradation. Collectively, the multifunctional design and preliminary biocompatibility of PDMI supported its further investigation as a potential intra-articular strategy for early PTOA intervention.

## 5 Conclusions

In summary, inspired by the Trojan horse concept, we developed a multifunctional vaccine-like nanoparticle, PDMI, that demonstrated prolonged intra-articular retention, enhanced hydration lubrication, ROS scavenging, and ROS-responsive ICA release. PDMI reduced mitochondrial oxidative stress, preserved mitochondrial membrane potential, alleviated inflammatory responses, and improved cartilage structural integrity and function-related ECM homeostasis in the 4-week rat PTOA model, as evidenced by reduced OARSI scores and MMP-13 expression together with increased Col2α expression. PDMI also exhibited favorable short-term biocompatibility and biosafety under the tested conditions. These findings supported further investigation of PDMI as a potential intra-articular strategy for early intervention in PTOA, while extended animal studies remain necessary to establish its long-term efficacy and safety.

## Ethics approval and consent to participate

The animal procedures were approved by the Animal Care and Welfare Committee of Peking University School of Stomatology (BDKQ-202510160923).

## CRediT authorship contribution statement

**Yinuo Yang:** Formal analysis, Investigation, Methodology, Writing – original draft. **Jinna Ren:** Formal analysis, Investigation, Methodology. **Gengtian Sun:** Formal analysis, Investigation, Methodology. **Keyi Huang:** Investigation, Methodology. **Xin Zhao:** Formal analysis, Supervision. **Aleksandr Poliakov:** Writing – review & editing. **Yuguang Wang:** Conceptualization, Funding acquisition, Supervision, Writing – review & editing. **Hongyu Zhang:** Conceptualization, Funding acquisition, Supervision, Writing – review & editing.

## Declaration of competing interest

The authors declare no conflict of interest.
