# Supplementary material for: Trojan horse-inspired vaccine-like nanoparticle with enhanced lubrication and active-passive synergistic anti-inflammation for post-traumatic osteoarthritis treatment
Source: Bioact Mater. 2026 Aug 12;67:415–36. doi: 10.1016/j.bioactmat.2026.08.005 (PMC13495601; doi:10.1016/j.bioactmat.2026.08.005)
Supplement: Supplementary file 1 [file mmc1.docx]

**Supporting information**

**Trojan Horse-Inspired Vaccine-Like Nanoparticle with Enhanced Lubrication and Active-Passive Synergistic Anti-Inflammation for Post-Traumatic Osteoarthritis Treatment**

**Figure S1**. ^1^H NMR spectrum of PDMI.

**Figure S2**. The element distribution of PDMI obtained from EDS.

**Figure S3**. The size distribution of PDMI under different pH conditions.

**Figure S4**. The UV-vis spectra of the released medium at different release times.

**Figure S5**. The image showing the color change of the PDMI solution over the course of drug release.

**Figure S6**. Friction curves of simulated synovial fluid with or without PDMI. (a)-(c) were the friction curves with a load of 1 N and frequencies of 1, 2, and 3 Hz, respectively.

(d)-(f) were the friction curves with a load of 2 N and frequencies of 1, 2, and 3 Hz, respectively.

**Figure S7**. Quantitative comparison of the COF of simulated synovial fluid with or without PDMI. The data were presented as mean ± SD, n = 3.

**Figure S8**. Quantitative comparison of the COF of PDMI with different concentrations. The data were presented as mean ± SD, n = 3.

**Figure S9**. Quantitative comparison of the COF of RPDMI with different concentrations. The data were presented as mean ± SD, n = 3.

**Figure S10**. Representative live/dead fluorescence images of chondrocytes cultured with PMI, PDM, or PDMI for 24 h.

**Figure S11**. Representative live/dead fluorescence images of macrophages cultured with PMI, PDM, or PDMI for 24 h.

**Figure S12**. Cellular uptake of PDMI after co-incubation.

**Figure S13**. Dual-fluorescence colocalization analysis of PDMI structural integrity.

**Figure S14**. The TEM image of macrophage exosome encapsulating nanoparticle.

**Figure S15.** Complement C3 opsonization enhanced the uptake of PDMI by RAW264.7 macrophages. The data were presented as mean ± SD, n = 3. ***p* < 0.01.

**Figure S16.** MA plot of DEGs.

**Figure S17.** Sankey diagram of DEGs and enriched KEGG pathways.

**Figure S18**. Chondrocyte viability under oxidative stress stimulation following different treatments. The data were presented as mean ± SD, n = 3. ns indicated no significant difference (*p* ≥ 0.05) and ***p* < 0.01.

**Figure S19.** Flow cytometric gating strategy for the identification of CD86-positive M1 and CD206-positive M2 macrophages within the F4/80-positive RAW264.7 cell population.

**Figure S20**. One-phase exponential decay fitting of the intra-articular fluorescence retention of Cy5-labeled PDMI and free Cy5. The data were presented as mean ± SD, n = 3.
